# BIRC5 as a master regulator in HCC: unraveling its role in tumor survival and therapeutic potential

**DOI:** 10.1007/s10142-025-01615-z

**Published:** 2025-06-05

**Authors:** Aya M. Ayoub, Elham Abdel-Badiea Mahmoud, Rania Hassan Mohamed, Mahmoud M. Tolba, Nahla S. Hassan, Mohamed Ghazy, Mahmoud ElHefnawi

**Affiliations:** 1 Biochemistry Department, Faculty of Science, Ain Shams University, Cairo, Egypt; 2 Clinical Research and Pharmaceutical Division, Egypt Ministry of Health and Population, Cairo, Egypt; 3 Biotechnology Program, Basic and Applied Science Institute, Egypt-Japan University of Science and Technology, New Borg El-Arab, Alexandria, Egypt; 4 Biomedical Informatics and Chemoinformatics Research Group, Informatics and Systems Department, National Research Center, Giza, Egypt

**Keywords:** Hepatocellular carcinoma, BIRC5, Knockout, CRISPR-Cas9 system

## Abstract

Hepatocellular carcinoma (HCC) is one of the most prevalent and lethal malignancies worldwide, with limited effective therapeutic options. Identifying novel targets is crucial for improving treatment strategies. In this study, we integrated bioinformatics and experimental approaches to uncover key regulatory genes in HCC. BIRC5 (survivin) emerged as a central hub gene, playing a pivotal role in apoptosis inhibition and cell cycle regulation. Using CRISPR-Cas9-mediated knockout in HepG2 cells, we demonstrated that BIRC5 depletion significantly suppressed cell proliferation and migration while inducing apoptosis. Furthermore, BIRC5 knockout led to cell cycle arrest, cytokinesis defects, and autophagy activation, highlighting its essential role in tumor maintenance. Functional assays, including colony formation, wound healing, flow cytometry, gene expression profiling, and transmission electron microscopy, validated these findings. Notably, the downregulation of key oncogenic pathways, including PI3K and AURKA, underscores the critical function of BIRC5 in sustaining HCC cell survival and proliferation. These results position BIRC5 as a promising therapeutic target, with the potential to disrupt tumor growth and metastasis. Targeting BIRC5 could offer a novel strategy for improving HCC treatment outcomes, paving the way for more effective therapeutic interventions against this aggressive cancer.

**Supplementary Information:**

The online version contains supplementary material available at 10.1007/s10142-025-01615-z.

## Introduction

Liver cancer stands as the third greatest cause of death due to cancer worldwide in 2022, with nearly 800,000 deaths per year, making it a significant global health burden (Bray et al. 2024). Hepatocellular carcinoma is a serious concern since it is the most frequently diagnosed type of primary liver cancer and the sixth most frequently encountered kind detected globally (Rashed et al. 2020). Despite advancements in research in medicine, the prognosis for HCC is still unfavorable since most patients are discovered at advanced stages due to the absence of noticeable symptoms (Forner et al. 2018). Currently, only 5% to 15% of HCC patients benefit from standard treatment approaches such liver transplantation, trans-arterial treatments, and surgical removal (Anwanwan et al. 2020). Additionally, HCC exhibits strong resistance to chemotherapy and immunotherapy, underscoring the urgent need for research to discover novel treatments (Cabral et al. 2020). In recent years, gene-editing technologies have emerged as promising tools for developing targeted therapies, with the potential of precisely modifying genes responsible for disease. The rapid advancement of genome engineering techniques has facilitated the correction of genetic abnormalities in cancer cells, both in laboratory and clinical settings. The clustered regularly interspaced short palindromic repeats (CRISPR)-associated protein Cas9, zinc-finger nucleases (ZFNs), and transcription activator-like effector nucleases (TALENs) are key gene-editing platforms. Among these, CRISPR-Cas9, which employs guide RNA (gRNA) to direct gene modifications, has shown exceptional effectiveness in various disease models, including cancer (Gaj et al. 2013; Niu et al. 2014). Our goal in this study was to use bioinformatics techniques to find a set of hub genes that might be used as therapeutic targets and biomarkers for HCC. According to our findings, BIRC5 is the most likely oncogene, underscoring both its potential as a target for therapy and its crucial role in the pathological process of HCC. This discovery lays the groundwork for future investigations into targeted therapeutics for HCC by illustrating the crucial function that BIRC5 plays in controlling cell survival and apoptosis.

Accumulating evidence has identified survivin as a crucial oncogene that is implicated in the development, progression, diagnosis, and management of liver cancer and other cancers (Kondapuram et al. 2023). Survivin, encoded BIRC5 gene (Baculoviral Inhibitor of Apoptosis Repeat Containing 5), related to the inhibitor of apoptosis (IAP) protein family and is involved in division of cells and apoptosis. Although it is almost invisible in healthy adult tissues, it is strongly expressed in the majority of malignancies (Altieri 2015). Notably, its expression increases up to 40-fold during the G2/M phase (Haglid et al. 1975), and it is strongly linked to key oncogenic processes, including tumor proliferation, metastasis, chemotherapy resistance, autoimmune diseases, poor prognosis, and reduced patient survival. These traits demonstrate BIRC5's value as a biomarker and its potential as a cancer treatment target (Li et al. 2021; Frazzi 2021).

BIRC5 has been identified as a significant contributor to tumor progression and development, chemoresistance, and metastasis through its participation in several signaling pathways, such as the JAK/STAT, Notch, PI3 K/AKT, TGF‐β, NF‐κ, Wnt/β‐catenin, Hippo pathways and p38/MAPK (Martínez-García et al. 2019). Various strategies, including the use of small‐molecule inhibitors, such as YM155 and FL188 (Wang et al. 2011; Ling et al. 2012); antisense oligonucleotides, such as LY2181308 and SPC3042 (Martínez-García et al. 2019); and small interfering RNAs (siRNAs) (Liu et al. 2013; Khan et al. 2016; Zhang et al. 2017) have been employed to knock out BIRC5 in cancer cells.

The CRISPR-Cas9 system offers a significantly specific, accurate, efficient in time, and cost-effective (Lone et al. 2018; Yang and Zhang 2023). While CRISPR-Cas9 has been widely utilized in cancer research (Alagoz and Kherad 2020; Rabaan et al. 2023; Jiang et al. 2020; Rajanathadurai et al. 2024) its applications in HCC remain relatively underexplored. Previous studies have revealed the success of CRISPR-Cas9 in targeting oncogenes such as GP73 (Liu et al. Jul. 2017), CD44 (Han et al. 2015), G9a (Wei et al. 2017), NCOA5 (He et al. 2018), CXCR4 (Wang et al. 2017), Sphk1 (Cai et al. 2017), eEF2 (Pott et al. 2017), NSD1 (Zhang et al. 2019), ASPH (Iwagami et al. 2016), mir-3188 (Zhou et al. 2017), HBsAg (Song et al. 2018), MECOM (Chen et al. 2024) and HBx (Rawal n.d.), all of which impair cell proliferation and migration. Additionally, CRISPR-Cas9 has been shown to effectively downregulate BIRC5 in several cancers, providing significant therapeutic benefits (Khan et al. 2016; Albadari and Li 2023; Zhao et al. 2017; Narimani et al. 2019).

As the first study to observe BIRC5 knockout in the HepG2 cell line using the powerful CRISPR-Cas9 technology to target BIRC5 specifically and assess the effects of its deletion in HepG2 cell lines we examined at how BIRC5 affected migration, apoptosis, cell proliferation, and cell cycle dynamic and our results indicate that targeting BIRC5 via CRISPR-Cas9 represents a potential clinical technique in HCC with the potential to open the door for more powerful anticancer therapies.

## Methods

**Fig. 1:**
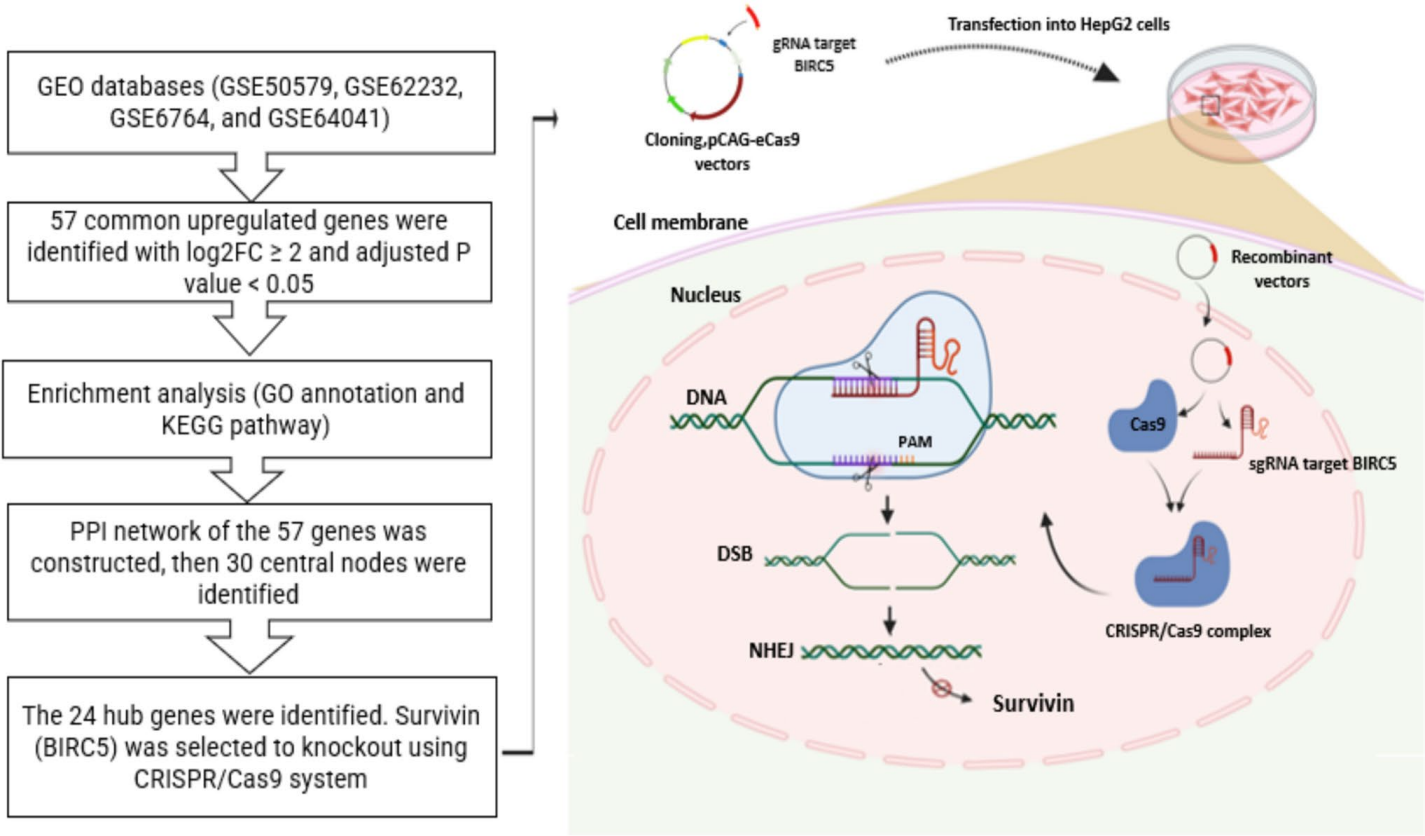
The workflow of bioinformatics analyses and the transfection process of the eCas9-BIRC5-gRNA recombinant plasmid. The CRISP-eCas9 system as a therapeutic approach in HCC through precise gene editing. The Cas9 enzyme, guided by the sgRNA, targets a specific genomic locus within the BIRC5 gene, resulting in the induction of a DSB. This DSB is repaired predominantly by the NHEJ pathway, introducing random insertions or deletions (indels) that cause frameshift mutations and subsequent gene knockout. (created by http://www.biorender.com/). GEO: Gene Expression Omnibus, GO: Gene ontology, KEGG: Kyoto Encyclopedia of Genes and Genomes, PPI: protein–protein interaction, Cas9: CRISPR-associated protein, BIRC5: baculoviral IAP repeat containing 5, DSB: double‐strand breaks, NHEJ: non‐homologous end‐joining

The flowchart of integrative bioinformatics and in vitro methodology used in the study is shown in Fig. 1.

- Bioinformatics studies (in silico)

### Microarray data and identification of commonly upregulated genes

The Gene Expression Omnibus (GEO) database was used to provide the gene expression profile information for HCC patients (https://www.ncbi.nlm.nih.gov/geo). The datasets used included GSE50579, GSE62232, GSE6764, and GSE64041, which comprised 61 HCC tissues and 7 normal hepatic tissues, 81 HCC tissues and 10 normal hepatic tissues, 27 HCC tissues and 10 normal hepatic tissues, and 60 HCC tissues and 5 normal hepatic tissues, respectively, as described by Zhang et al. (Zhang, et al. 2018). To identify genes that were significantly elevated in HCC tissues, the GEO2R web tool (https://www.ncbi.nlm.nih.gov/geo/geo2r/) was utilized to compare expression levels between HCC and normal tissues in each dataset. Significantly upregulated genes with log2 FC $\ge$ 2 and adjusted P value < 0.05 were identified in each dataset (Fu et al. 2021). The intersecting upregulated genes from all four GEO datasets were recognized via Venn diagram analysis.

### Functional enrichment analysis

To investigate the biological roles and pathways associated with the commonly upregulated genes, we utilized the Database for Annotation, Visualization, and Integrated Discovery (DAVID) (http://david.ncifcrf.gov), a web-based platform. Gene ontology (GO) function analysis was performed, categorizing genes into biological processes (BP), cellular components (CC), and molecular functions (MF). Additionally, Kyoto Encyclopedia of Genes and Genomes (KEGG) pathway analysis was conducted to identify significantly enriched pathways. A statistical threshold of P-value <0.05 was applied to determine significant findings.

### Protein‒protein interaction (PPI) network construction and module analysis

To explore gene interactions and their functional relevance, a protein–protein interaction (PPI) network was generated using the Search Tool for the Retrieval of Interacting Genes (STRING). This allowed for the identification of potential relationships among the upregulated genes. The network visualization and further analysis were conducted using Cytoscape software v3.6.1, facilitating a clearer representation of these molecular interactions (Fu et al. 2021; Mi et al. 2020). It was established which central nodes were related to HCC. These key nodes were analyzed using GO and KEGG, and Cytoscape was employed to show the results. To identify the hub genes in the PPI network, the cytoHubba plugin was applied to select the most upregulated genes. As hub genes, the top 23 significant upregulated genes were chosen.

### Validation of the expression levels and overall survival (OS) analysis of the top three hub genes

The mRNA expression levels of the three highest-ranking hub genes were validated through the Gene Expression Profiling Interactive Analysis (GEPIA) online tool (http://gepia2.cancer-pku.cn/), which utilizes both HCC and normal hepatic tissues for comparison. Additionally, the correlation between these hub genes and overall survival (OS) was assessed through the GEPIA database to evaluate their prognostic significance.

### Design of gRNA and formation of the pCAG-eCas9-gRNA CRISPR recombinant vector

The CRISPR-Cas9 vector used in this study was obtained from Addgene (catalog #79,145, Cambridge, MA). Two single guide RNAs (sgRNAs) were designed with BbsI restriction sites via the CHOPCHOP web tool (http://chopchop.cbu.uib.no/). Sequences were selected to minimize off-target mismatches in the human genome while ensuring that they contained protospacer adjacent motifs (PAMs) of the form 5′-NGG-3′. These sgRNAs were subsequently cloned and inserted into the pCAG-eCas9 CRISPR vector, which specifically targets the opposite strands at exon 1 of the BIRC5 gene. As a control, a negative (scramble) vector was constructed. The correct insertion of gRNAs at the intended positions and in the appropriate orientation was validated via colony PCR assays and Sanger sequencing at the gRNA scaffold site of the recombinant vectors. The constructed gRNA sequences were as follows: Oligo gRNA1 (5′-GATGCGGTGGTCCTTGAGAAA-3′) and Oligo gRNA2 (5′-GCAAGTCTGGCTCGTTCTCAG-3′).

### Cell culture and transfection

HepG-2 cell line was acquired from VACSERA-Cell Culture Unit (Dokky, Giza, Egypt). The cell lines were suspended in RPMI 1640 medium (Sigma-Aldrich, St. Louis, MO, USA) that contained 10% fetal bovine serum (FBS), inactivated at 56 °C for 30 min, (Sigma-Aldrich, St. Louis, MO, USA) and 1% penicillin/streptomycin (Sigma-Aldrich, St. Louis, MO, USA) in a fully saturated atmosphere at 5% CO_2_ and 37 °C until 70–80% confluence. After being seeded at a cell density of 1.5 × 10^5^ cells/well on a 6-well plate, the HepG2 cells were incubated overnight. A recombinant eCas-BIRC5-gRNA vector was introduced into Opti-MEM via Lipofectamine 3000 (Invitrogen, Carlsbad, CA, USA) for transfection according to directions provided by the manufacturer. The transfected empty eCas vector was utilized as the scramble sample. After 48 h, the media were discarded, and the transfected cells were visualized via a Leica DMI 8 confocal microscope (Leica Microsystems, Mannheim, Germany). The fluorescence intensity was subsequently valued via Leica DMI 8 software, and the levels of the green fluorescent protein (GFP) signal were subsequently determined.

### Quantitative real-time PCR (RT‒qPCR)

As directed by the manufacturer, TRIzol™ Reagent (Invitrogen, Carlsbad, CA, USA) was handled to extract RNA from HepG2 cells (Chomczynski 1993). The RevertAid™ First-Strand cDNA Synthesis Kit (Thermo Fisher Scientific, Waltham, MA, USA) synthesized cDNA, and RevertAid Reverse Transcriptase was incubated for 70 min at 5 °C. RT-qPCR was on the Step One Plus Real-Time PCR System (Applied Biosystems, USA) with the Maxima SYBR Green qPCR Kit (Thermo Fisher Scientific, Waltham, MA, USA). The forward primer: 5ʹ–GCCAGATGACGACCCCATAG − 3ʹ, and the reverse primer: 5ʹ–GCGCACTTTCTTCACAGTTTC − 3ʹ, were unique to BIRC5. Relative gene expression was estimated using the 2-ΔΔCt approach, and gene expression levels were standardized internally using GAPDH as a reference (Livak and Schmittgen 2001) whose reliable was confirmed by an online computational program RefFinder (Xie et al. 2023). The experiment was performed in triplicate with biologically independent replicates.

#### Western blot

Western blotting was accomplished to examine the expression of the BIRC5 protein. Briefly, total protein was extracted from transfected cells 72 h after transfection, utilizing RIPA cell lysis buffer (Thermo Fisher Scientific, Waltham, MA, USA), and the BSA technique was utilized to quantify the amount of protein (Kurien and Scofield 2003). A 15% SDS-PAGE gel was used to resolve approximately 30 µg sample protein for each sample, which was subsequently electroblotted onto a polyvinylidene difluoride (PVDF) membrane. The membrane was incubated for an entire night at 4 °C using a primary antibody specific to BIRC5, with GAPDH acting as the loading control. Following incubation, the membrane was washed and treated for 1 h at room temperature with secondary antibodies coated with horseradish peroxidase (HRP) in PBS-Tween. Protein bands were found using an upgraded chemiluminescence detection kit (Thermo Fisher Scientific, Waltham, MA, USA). The experiment was performed in a triplicate with biologically independent replicates.

### Colony formation assay

Colony formation assays were performed following the protocol described by Franken et al. (Franken et al. 2006). For both treated and control groups, 3 × 10^3^ HepG2 cells were plated per well in a 6-well plate. On day 10, after being rinsed with cold PBS, the cells were fixed using a 1:7 acetic acid to methanol solution. Following fixation, the colonies were stained with a 0.5% crystal violet (Sigma-Aldrich, St. Louis, MO, USA) solution, then imaged for analysis. The experiment was performed in triplicate with biologically independent replicates.

### Cell cycle assays

Utilizing flow cytometry, the cell cycle was examined. In short, HepG2 cells, both treated and control, were fixed for 1 h at 4 °C in 60% ethanol. An ACEA NovocyteTM flow cytometer (ACEA Biosciences Inc., San Diego, CA, USA) was used to evaluate the cells after they had been marked with propidium iodide (PI) (Sigma-Aldrich, St. Louis, MO, USA) at room temperature for 15 min in the dark. Multivariate data interpretation was conducted by quadrant segmentation analysis with ACEA NovoExpressTM processing tools (ACEA Biosciences Inc., San Diego, CA, USA) to ascertain the proportion of cells in each cell cycle phase. The experiment was performed in duplicate with biologically independent replicates.

In this study, the expression levels of several critical cell cycle regulatory genes, such as the cyclin-dependent kinases CDK1, CDK2, and AURKA as well as the checkpoint regulators P21 and P53, were quantified via RT‒qPCR to further investigate the impact of the treatments on cell cycle progression. Additionally, the expression of PI3 K, a crucial oncogene linked to HCC pathways associated with proliferation of cells, autophagy, and migration, was also evaluated via RT‒qPCR, as outlined in previous methods. The experiment was performed in duplicate with biologically independent replicates.

### Apoptosis assays

After 72 h of transfection, apoptosis quantification was performed by flow cytometry. Following dual staining with 5 µL each of FITC-Annexin V and PI during a 15 min dark incubation at room temperature, apoptotic cell percentages were determined using quadrant analysis in ACEA NovoExpressTM software (ACEA Biosciences Inc., USA). As described in earlier techniques, RT-qPCR was used to assess the expression levels of important apoptotic markers, including Caspase 8 and the antiapoptotic marker Bcl2, to further validate apoptosis. The experiment was performed in duplicate with biologically independent replicates.

### Wound healing assay

Cell migratory capacity was assessed using a scratch wound assay. HepG2 cells were plated in 6-well culture plates (1.5 × 10^5^ cells/well) containing 3 ml of complete growth medium and allowed to adhere for 24 h prior to wound induction (Liang et al. 2007). The cells were then transfected with either the eCas vector or eCas-BIRC5-gRNA vectors. A pipette tip was used to simulate a wound and generate a scratch once the cell monolayers had reached about 90% confluence. When the cell monolayers reached approximately 90% confluence, a scratch was created using a pipette tip to mimic a wound. The cells were cultured under standard conditions, and the wound closure was detected and measured at 0, 24 and 48 h. The experiment was performed in triplicate with biologically independent replicates.

### Transmission electron microscopy (TEM)

Seventy-two hours post-transfection, following the method outlined by Rao et al. (Rao et al. 2010), both eCas-BIRC5-gRNA-transfected HepG2 cells and untreated HepG2 cell pellets were subjected to PBS washing followed by primary fixation in chilled 2.5% glutaraldehyde solution. Post-PBS rinsing, samples underwent secondary fixation using 1% OsO₄ for 60 min. Dehydration was carried out using a graded ethanol series (50%, 70%, and 90%) for 15 min at each concentration, followed by absolute ethanol for 20 min and acetone for an additional 20 min. The dehydrated cells were then embedded in Epon 812 resin (Shell Chemical Co., San Francisco, CA, USA). Ultrathin Sects. (90 nm) were prepared, mounted on copper grids, and stained with 0.6% uranyl acetate and 0.4% lead citrate for 3 min each. Images were acquired using a JEM-1200EX electron microscope.

## Statistical analysis

Statistical comparisons between two experimental groups were conducted using Student's t-test, while one-way analysis of variance (ANOVA) was applied for comparisons among three or more groups. All analyses were performed using GraphPad Prism version 8.0 (GraphPad Software, San Diego, CA), with a significance level of p < 0.05.

## Results

### Identification of significantly upregulated genes in HCC

In all 356, 94, 135, and 46 upregulated genes were linked from the GSE50579, GSE62232, GSE6764, and GSE64041 datasets, respectively, via the GEO2R online tool. The Venn diagram **(**Fig. S1) illustrates the intersecting genes among these four datasets. Notably, 46 genes were found to be commonly upregulated across three of the datasets, whereas only 11 genes were commonly upregulated across all four datasets **(**Table 1).

**Table 1:** Common upregulated expressed genes among the four GEO datasets

Datasets	Total	Elements
GSE50579 GSE62232 GSE64041 GSE6764	11	LEF1, PRKAA2, CAP2, DTL, EDIL3, GPC3, ROBO1, VWF, SULT1 C2, ACSL4, KRT23
GSE50579 GSE62232 GSE6764	39	PDZK1IP1, FAM83D, CCNB1, ASPM, FLVCR1, HMMR, GINS1, CCL20, ANLN, BIRC5, SQLE, KIF20 A, ITGA6, COL15 A1, PRC1, CDK1, TYMS, PLVAP, RACGAP1, HKDC1, CTHRC1, UHRF1, RRM2, ZWINT, NDC80, TOP2 A, KIAA0101, CCNA2, TTK, CDKN3, PBK, NEK2, AURKA, UBE2 T, CRNDE, NUSAP1, BUB1B, MAD2L1, DLGAP5
GSE50579 GSE62232 GSE64041	1	ANXA2
GSE62232 GSE64041 GSE6764	6	SPINK1, FAT1, AKR1B10, SPP1, LCN2, DTNA

### Functional enrichment analysis

KEGG pathway enrichment analysis determined that the commonly upregulated genes were enhanced in five pathways: the cell cycle, progesterone-mediated oocyte maturation, ECM-receptor interaction, oocyte meiosis and the p53 signaling pathway. These genes were enriched in 101 GO terms. A detailed description of the KEGG pathway and the top 15 enriched GO terms of the upregulated genes are shown in Table S1 (supplementary information (SI)).

### PPI and modular analysis

The PPI network of the commonly upregulated genes was constructed via the STRING database, resulting in a network composed of 56 nodes and 445 edges, with a local clustering coefficient of 0.765 and a PPI enrichment *p*-value < 1.0e-16 (Fig. S2a). In addition, 30 central nodes were identified and are shown in Fig. S2 b. The KEGG analysis of these 30 central genes revealed significant enrichment in pathways such as the cell cycle, progesterone-mediated oocyte maturation, oocyte meiosis, the p53 signaling pathway, cellular senescence, pyrimidine metabolism, progesterone-mediated oocyte maturation and human T-cell leukemia virus 1 infection (HTLV-I). The hub genes were identified via the cytoHubba plugin. The top 23 hub genes identified across the HCC datasets are listed in Table SI2). Among these genes, the BIRC5 gene was identified as having the highest degree score of 60, indicating that it is a key candidate for further investigation.

### Validation of the expression levels and overall survival (OS) analysis of the top three hub genes

As shown in Fig. S3 a, the GEPIA online tool results showed that the top three hub genes (BIRC5, HMMR, and KIF20 A) had significantly higher mRNA expression levels in HCC patients. Furthermore, the OS plots (Fig. S3 b) showed that serious overall survival was linked with higher expression levels of BIRC5, HMMR, and KIF20 A, suggesting that these genes may be used as prognostic and therapeutic markers for HCC (supplementary material).

### CRISPR-Cas9 system transfection and Knockout of BIRC5 expression in HepG2 cells

**Fig. 2:**
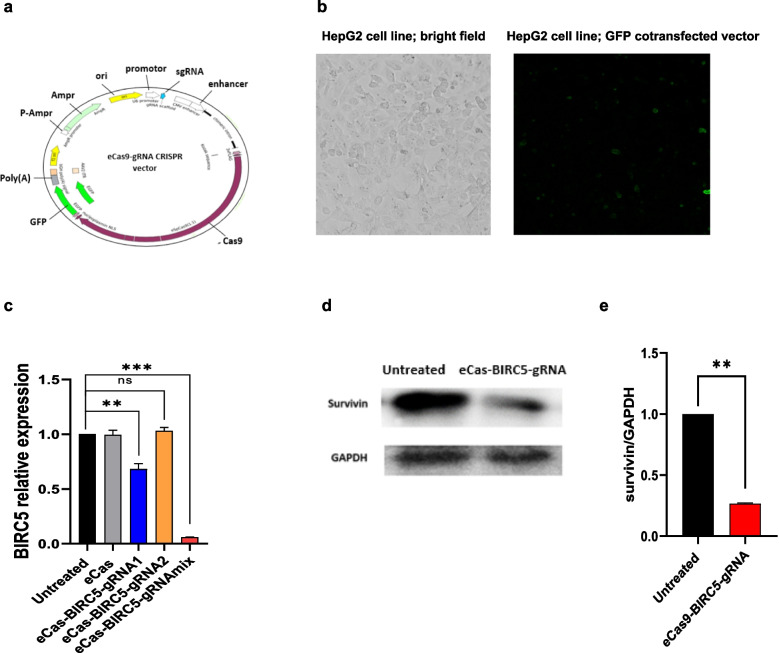
Transfection and knockout BIRC5 expression of HepG2 cells by CRISPR-Cas9 system. **a**) Structure of eCas9-gRNA CRISPR vector. **b**) Representative images of GFP expression mediated by pCAG-eCas9 vectors in transfected HepG2 cells. Bar = 100 µm. **c**) Quantitative evaluation of BIRC5 expression in HepG2 cell lines via qRT-PCR 72 h after transfection. Data is represented as mean ± SD), ** *p* < 0.01, *** *p* < 0.001. **d**) Expression of BIRC5 normalized to GAPDH in transfected HepG2 cells by Western blot analysis. **e**) A histogram showing the changes in the BIRC5 expression in transfected HepG2 cells with eCas9-gRNAmix, compared to untreated control group. Data is represented as mean ± SD, n = 3, ** *p* < 0.01

A map of the recombinant plasmid containing Cas9, GFP, and sgRNA transcription components is shown in Fig. 2a. Transfection of the eCas-BIRC5-gRNA plasmid into HepG2 cells was successful, as shown in Fig. 2b, where GFP expression was used as a visual indicator of transfection efficiency. The fluorescence intensity, measured via Leica software, was approximately 10.833 arbitrary units (au) in eCas-transfected cells, 15.575 au in eCas-BIRC5-gRNA1-transfected cells, and 16.622 au in eCas-BIRC5-gRNA2-transfected cells.

Expression analysis of BIRC5 mRNA was performed in HepG2 cells transfected with eCas-BIRC5-gRNA1, gRNA2, and a gRNAmix in comparison to the control groups (eCas and untreated HepG2 cells). The null vector (eCas) produced no significant changes in BIRC5 expression due to the absence of gRNA targeting BIRC5 sequences. In contrast, transfection with eCas9-BIRC5-gRNA1 resulted in a significant fourfold decrease in BIRC5 expression. However, eCas9-BIRC5-gRNA2 transfection did not significantly differ from that in the control groups. Notably, cells transfected with the eCas9-BIRC5-gRNAmix exhibited highly significant 14-fold downregulation of BIRC5 expression (Fig. 2c), leading to its selection for further studies.

The knockout of BIRC5 in HepG2 cells transfected with eCas9-BIRC5-gRNAmix was further confirmed through Western blot analysis, with survivin expression normalized to that of GAPDH. Figure 2d-e demonstrates a significant 73 ± 3% reduction in BIRC5 protein levels in eCas9-BIRC5-gRNAmix-transfected cells compared with those in the control group.

### Knockout of BIRC5 reduces HepG2 cell growth and induces cell cycle arrest

**Fig. 3:**
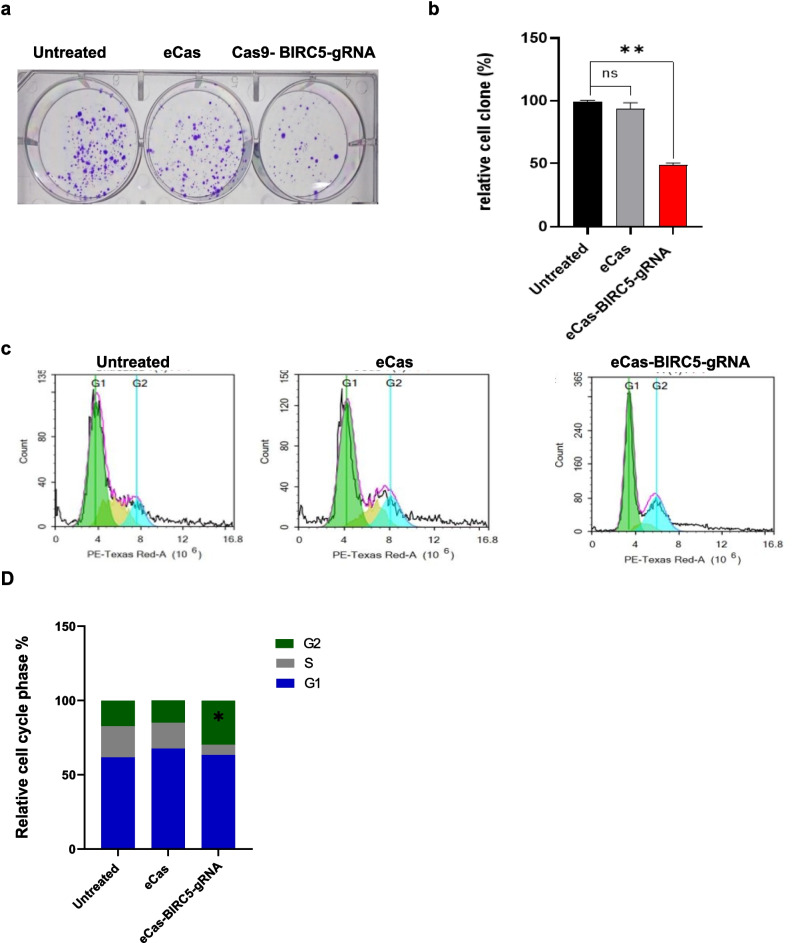
Representative images of the clonogenic assay and flowcytometric analysis of cell cycle demonstrated the proliferation ability of HepG2 cells. **a**) Images of the cell clones formed from dissociated HepG2 cells after two weeks of incubation. **b**) Histogram showing the difference between the samples in cell proliferation. Data is represented as mean ± SD, *n* = 3, * *p* < 0.05, ** *p* < 0.01. **c**) Cell cycle analysis of transfected HepG2 cells image with PI staining and using NovoExpress 1.1.0 Software. d) Histogram demonstrates the changes in cell cycle phases with eCas-BIRC5-gRNAmix treatment. Data is represented as mean ± SD, *n* = 2, * *p* < 0.05

In the colony formation assay, compared with untreated HepG2 cells, eCas-BIRC5-gRNA-transfected HepG2 cells presented a 50 ± 3% reduction in colony number (Fig. 3a-b). These findings suggest a significant reduction in cell growth following BIRC5 knockout. To further explore the consequences of BIRC5 knockout on cell cycle progression, flow cytometric cell cycle analysis was conducted on eCas9-BIRC5-gRNA-transfected HepG2 cells. The results revealed a substantial increase in the cell population at G2/M phase, showing cell cycle arrest (Fig. 3c-d). Specifically, the proportion of cells in the G2 phase was significantly greater in the eCas9-BIRC5-gRNA-transfected group than in the control group.

**Fig. 4:**
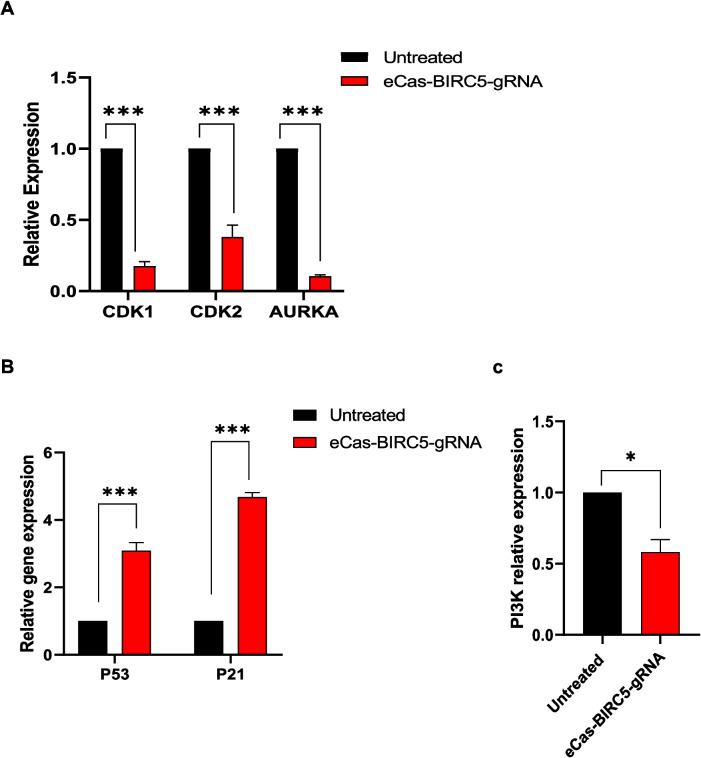
Quantitative of cell cycle markers and PI3 K gene in transfected HepG2 cell compared with untreated cells to detect the effects of downregulation BIRC5 in cell cycle and cancer pathways. **a**) Relative expression of cell cycle regulators markers (CDK1 & CDK2) and AURORA. b) Relative expression of checkpoint gene expression (P21 & P53). c) Relative expression PI3 K gene. Data is represented as mean ± SD, *n* = 2, * *p* < 0.05, ** *p* < 0.01, *** *p* < 0.001

These findings were corroborated by RT‒qPCR analysis, which revealed a marked decrease in the expression of cell cycle regulators. eCas9-BIRC5-gRNA transfected cells showed 8.5-fold and 5.5-fold decreases in CDK1 and CDK2 mRNA levels, respectively, in comparison to the untreated group (Fig. 4a). In contrast to other observations, we detected significant elevation in transcriptional levels of the tumor suppressor genes P53 and P21 in transfected cell populations (Fig. 4b), further confirming the induction of G2/M phase arrest.

AURKA serves as a critical regulator of mitotic progression, cell cycle control, and oncogenic pathway activation. We evaluated AURKA expression in HepG2 cells transfected with eCas-BIRC5-gRNA in order to examine the connection between BIRC5 and AURKA. After BIRC5 deletion, our data confirmed a significant decrease in AURKA gene expression (Fig. 4a). Additionally, it is known that BIRC5 activates the PI3 K/AKT pathway, which is necessary for metastasis, autophagy, and cell survival. We examined the levels of PI3 K mRNA expression in HepG2 cells transfected with eCas9-BIRC5-gRNA in order to learn more about how BIRC5 affects this pathway (Fig. 4c). According to the data interpretation, BIRC5 depletion adversely impacts both the AURKA and PI3 K signaling pathways, as evidenced by the much lower AURKA and PI3 K expression in BIRC5 mutant cells compared to untreated control cells. These results imply that BIRC5 is essential for controlling carcinogenic pathways that are vital for the development of HCC.

### Knockout of BIRC5 induces apoptosis

**Fig. 5:**
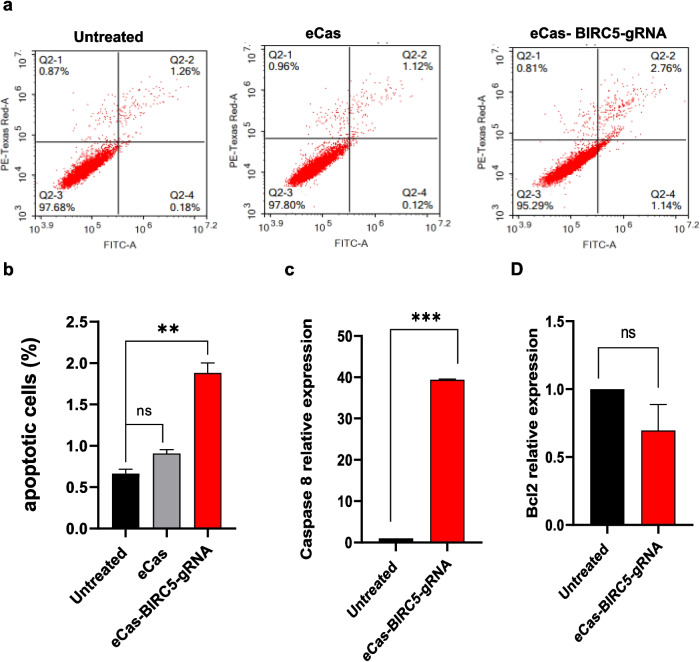
Flowcytometric analysis of Apoptosis in HepG2 cells and Apoptotic markers gene expression. **a**) Images of flowcytometric results by Software: NovoEepress 1.1.0. **b**) Histogram showing the difference between the samples in cell apoptosis. Data is represented as mean ± SD, ** *p* < 0.01. c&d) Histogram of Caspase 8 and Bcl2 gene expression levels in HepG2 cells using RT-qPCR normalized by GAPDH and compared eCas-BIRC5-gRNAmix transfected cells with untreated (control). Data is represented as mean ± SD, *n* = 2, * *p* < 0.05, ** *p* < 0.01, *** *p* < 0.001

The induction of apoptosis resulting from BIRC5 knockout in HepG2 cells was assessed via flow cytometry, complemented by analyses of the mRNA expression levels of key apoptotic markers, as depicted in Fig. 5. Flow cytometric analysis showed a significant shift in the number of transfected cells toward the late apoptosis phase (Q4), indicating a strong apoptotic response following BIRC5 disruption. These data indicate that the knockout of BIRC5 effectively triggers apoptotic pathways in HepG2 cells, as shown in Fig. 5a-b. To further validate these findings, RT‒qPCR was used to measure the expression of key apoptotic markers. The results revealed an ~ 39.5-fold increase in Caspase 8 expression in eCas-BIRC5-gRNA-transfected cells, confirming the activation of apoptosis. However, no discernible alteration in the expression of the Bcl2 gene was observed in the transfected cells compared with the untreated control cells (Fig. 5c-d).

### The Knockout of BIRC5 suppresses the migration of HCC cells

**Fig. 6:**
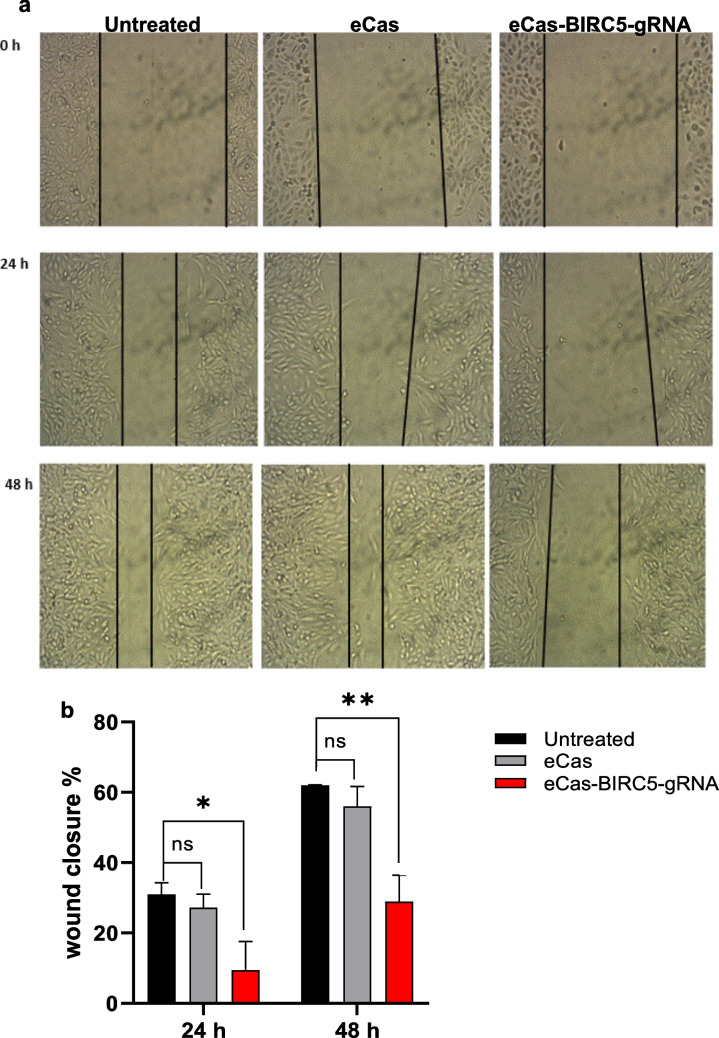
Effects of eCas-BIRC5-gRNA on the migration of HepG2 cells. **a**) Representative images of wound healing assays of HepG2 cells in 0 h, 24 h and 48 h after scratch was occurred (magnification power = 10 X). **b**) Bar diagram showing relative wound closure (%) in transfected eCas9-gRNA cells compared with control groups. Data is represented as mean ± SD, *n* = 3, * *p* < 0.05, ** *p* < 0.01

The migratory capacity of eCas-BIRC5-gRNA-transfected HepG2 cells was assessed using a scratch wound assay. Quantitative analysis revealed significantly reduced wound closure rates of 10 ± 5% at 24 h and 29 ± 5% at 48 h post-wounding, demonstrating impaired cell motility following BIRC5 knockout. In contrast, the control groups, which included untreated HepG2 cells and those transfected with the eCas null vector, presented wound closure percentages of 30 ± 3% and 60 ± 2%, respectively (Fig. 6). These findings revealed significant impairment of wound closure in the BIRC5 knockout cells compared with the untreated HepG2 cells at both 24 h and 48 h, suggesting that the knockout of BIRC5 effectively inhibits cell migration.

### TEM images revealed apoptosis, cytokinesis defects, and autophagy in BIRC5 knockout HepG2 cells

**Fig. 7:**
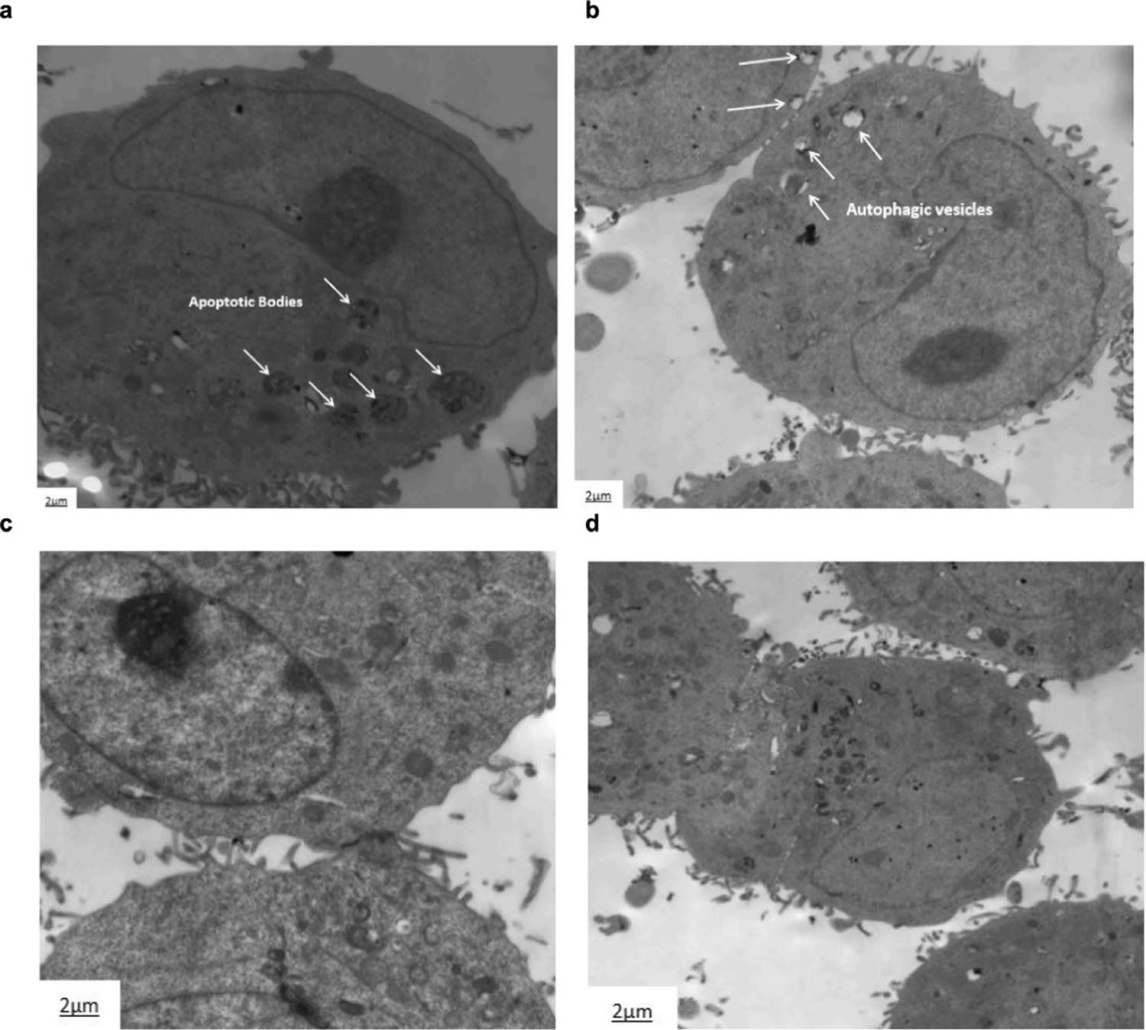
Transmission electron microscopy images of HepG2 cells before and after KO BIRC5. **a**) eCas-BIRC5-gRNA transfected HepG2 cells represented apoptotic bodies. **b**) Autophagic vesicles in eCas-BIRC5-gRNA transfected HepG2 cells were evaluated by electron microscopy. **c**) Normal HepG2 cell division represents the connection between the daughter cells and midbodies were formed. **d**) eCas-BIRC5-gRNA transfected HepG2 cells had disrupted cell connection during cytokinesis between daughter cells. *n* = 2, Bars: 2 μm

To further investigate the effects of BIRC5 knockout on cytokinesis and its role in cell cycle arrest at the G2 phase, TEM was employed to examine eCas-BIRC5-gRNA-transfected HepG2 cells. TEM analysis revealed clear signs of apoptosis, characterized by the presence of apoptotic bodies and autophagic vesicles, as shown in Fig. 7a-b. Additionally, BIRC5 knockout cells presented defects in cytokinesis, with incomplete telophase stages and disrupted cell division processes (Fig. 7d). In BIRC5-depleted HepG2 cells, failure to form midbodies resulted in an inability to establish the cleavage furrow required for successful cytokinesis between daughter cells.

## Discussion

The third most common cause of cancer-related death is HCC. Despite progress in treatment options, the overall prognosis for HCC patients remains unfavorable, underscoring the critical need for identifying novel therapeutic targets (Vogel and Saborowski 2020). To identify hub genes in HCC patients that have a high predictive potential for the disease, we used bioinformatics analyses in this work. Among these genes, BIRC5 (survivin) was identified as the top candidate hub oncogene, with a degree score of 60, prompting our focused investigation into its role in HCC progression.

BIRC5 is widely recognized for its positive association with cell survival, drug resistance, and cancer metastasis. It promotes cell proliferation and invasion while suppressing apoptosis and cell cycle arrest (Wheatley and Altieri 2019; Su 2016). Notably, BIRC5 is significantly overexpressed in most malignant tumors but is absent or minimally expressed in normal tissues, making it a highly talented target for cancer therapy (Martínez-García et al. 2019). Consistent with our bioinformatics analysis, Wang et al. (Wang et al. 2020) identified BIRC5 as the central hub gene in HCC using advanced bioinformatics techniques. Previous research has highlighted the pivotal role of BIRC5 in HCC and its potential for targeted cancer treatment (Kondapuram et al. 2023; Frazzi 2021; Albadari and Li 2023). In our study, we explored the consequences of BIRC5 knockout in the HepG2 cell line using the CRISPR-Cas9 system. This gene-editing technology is among the most powerful tools for gene therapy and has been successfully applied in the treatment of various cancers, demonstrating broad therapeutic potential (Alagoz and Kherad 2020). By targeting BIRC5, we aimed to uncover its mechanisms in HCC progression and assess its feasibility as a therapeutic target.

Our findings revealed that BIRC5 knockout via the CRISPR-Cas9 system markedly suppressed cell proliferation and triggered apoptosis, as evidenced by colony formation and cell cycle assays. Specifically, the proliferation and colony formation capacity of HepG2 cells were reduced by 50 ± 3% in eCas-BIRC5-gRNA-transfected cells compared to untreated controls. Cell cycle arrest at the G2 phase and a decrease in the total number of cells contributed to this decrease in proliferation, underscoring the crucial function of BIRC5 in controlling mitosis (Fig. 3). BIRC5 is a critical member of the chromosomal passenger complex (CPC), where it modulates microtubule dynamics through its interaction with tubulin (Albadari and Li 2023). Our results indicate that BIRC5 knockout causes a blockage at the G2/M phase and impaired cytokinesis in treated cells. This conclusion is further supported by the TEM images shown in Fig. 7d, which reveal cytokinesis failure and disruption of the midbodies connecting daughter cells.

These findings align with those of Yang et al. (Yang et al. 2015), who reported that BIRC5 knockout using siRNA in human sacral chondrosarcoma significantly reduced the G2/M phase population in SW1353 cells and influenced CDK2 activity. In our study, we observed an eightfold and sixfold reduction in the mRNA expression levels of CDK1 and CDK2, respectively, in eCas-BIRC5-gRNA-transfected HepG2 cells (Fig. 4a). This supports previous studies showing that CDK1 interacts with BIRC5 to promote phosphorylation, thereby conferring antiapoptotic properties (Chandele et al. 2004). Additionally, Suzuki et al. (Suzuki et al. 2000) demonstrated that BIRC5 interacts with Cdk4 to activate the Cdk4/Cyclin D1 and Cdk2/Cyclin E complexes, accelerating the G1/S phase transition, inducing resistance to G1 arrest, and increasing the S phase population. This interaction also suppresses the p53 and p21 checkpoint genes (Ito et al. 2000). p21, a transcriptional target of p53, functions as an inhibitor of cyclin-dependent kinases (CDKs), leading to cell cycle suppression (Engeland 2022). In line with these observations, we found a 4.6-fold increase in p21 gene expression in BIRC5 knockout HepG2 cells. Similarly, we noted a threefold increase in the expression of the p53 tumor suppressor gene in eCas-BIRC5-gRNA-transfected cells (Fig. 4b). p53 regulates numerous pathways, including cell cycle arrest, DNA repair, apoptosis, autophagy, and metabolism, to prevent tumorigenesis (Wang et al. 2023). Correspondingly, Wang et al. (Wang et al. 2004) demonstrated that BIRC5 modulates p53 activity, showing that BIRC5 disruption in BaF3 cells treated with Adriamycin led to decreased Mdm2 levels and increased p53 protein expression.

To our knowledge, this study is the first to reveal the impact of BIRC5 knockout on AURKA gene expression. We observed a significant tenfold reduction in AURKA gene expression in eCas-BIRC5-gRNA-transfected HepG2 cells compared to untreated cells (Fig. 4a). AURKA was selected for its critical involvement in various mitotic processes closely linked to BIRC5, such as the formation of mitotic spindle microtubules, centrosome duplication and maturation, chromosomal alignment, spindle assembly checkpoint regulation, and cytokinesis. Kamran et al. (Kamran, et al. 2017) demonstrated that AURKA stabilizes BIRC5 in gastric cancer by targeting FBXL7. Our results indicate that BIRC5 knockout suppresses AURKA, leading to cytokinesis failure and midbody disruption, as evidenced by TEM images of treated HepG2 cells.

Growing evidence suggests that BIRC5 knockout triggers both apoptosis and autophagy (Wang et al. 2011; Cheung et al. 2020). In our study, we found that BIRC5 knockout stimulated apoptosis, as demonstrated by Annexin V staining through flow cytometry and a 39.5-fold increase in caspase-8 mRNA expression (Fig. 5). This observation is consistent with the known function of BIRC5 in inhibiting caspases and suppressing apoptosis. Further confirmation was provided by the presence of apoptotic bodies in eCas-BIRC5-gRNA-transfected HepG2 cells, as visualized by electron microscopy (Fig. 7a). However, BIRC5 knockout did not significantly alter the anti-apoptotic gene Bcl-2 in HepG2 cells. Feng et al. (Feng et al. 2013) reported that inhibiting BIRC5 via YM155 in leukemia HL-60 and U937 cell lines promoted apoptosis, significantly elevating caspase-3 and caspase-8 levels while reducing Mcl-1 expression, with no change in Bcl-2 levels. BIRC5 inhibits apoptosome formation and procaspase-9 activation, thereby preventing apoptosis (Peery et al. 2017). Additionally, the BIRC5-apoptosis-inducing factor (AIF) complex protects cells from apoptotic stimuli that induce DNA fragmentation and chromatin condensation (Li et al. 2021; Albadari and Li 2023).

Autophagy, a form of type II programmed cell death, serves as a fundamental regulator of maintaining intracellular quality control. It exerts antitumor effects and is involved in multiple signaling pathways, thereby inhibiting the initiation and progression of HCC (Liu et al. 2017). Our TEM analysis revealed the presence of autophagic vesicles in eCas-BIRC5-gRNA-treated HepG2 cells (Fig. 7b), indicating that BIRC5 knockout effectively induced autophagy in these cells. Mechanistically, BIRC5 suppresses autophagy by interfering with the formation of autophagosomes (Lin et al. 2020). Furthermore, Huang et al. (Huang et al. 2013) and Wang et al. (Wang et al. 2011) demonstrated that silencing BIRC5 using siRNA in various cell lines activated autophagy. This was supported by the inhibition of the XIAP-Mdm2-p53 complex and the transformation of the autophagosomal marker protein LC3 from its cytosolic form (LC3I) to its membrane-bound form (LC3II). The accumulation of LC3II is a hallmark of integral autophagic flux and is consistent with autophagy induction. Additionally, previous studies have identified the PI3 K/AKT/mTOR signaling pathway as a key regulator of autophagy suppression in HCC (Liu et al. 2017). Our findings revealed that BIRC5 knockout significantly reduced PI3 K gene expression by fivefold, potentially contributing to the induction of autophagy in eCas-BIRC5-gRNA-transfected HepG2 cells by restraining the PI3 K/AKT/mTOR pathway.

Our findings revealed that BIRC5 knockout significantly suppressed cell migration. The wound closure rate was significantly reduced in eCas-BIRC5-gRNA-transfected HepG2 cells compared to untreated cells at both 24 and 48 h (Fig. 6). Additionally, BIRC5 knockout inhibited the expression of PI3 K (Fig. 4c), a key player in EMT and angiogenesis (Peng et al. 2022). Prior investigations have established that BIRC5 enhances angiogenesis and migration in HCC cells (Su 2016). Yu et al. (Yu et al. 2021) demonstrated that BIRC5 is associated with metastasis, invasion, and EMT by modifying WNT/β-catenin signaling. Furthermore, Tai et al. (Tai et al. 2012) and Zhao et al. (Zhao et al. 2017) reported that BIRC5 knockout suppresses EMT through upregulating the epithelial marker cytokeratin 7 and downregulating mesenchymal markers such as β-catenin, vimentin, and snail2. Recently, a comprehensive review has been published on the role of survivin in HCC (Mohamed et al., 2025).

## Conclusions

This study demonstrates that BIRC5 deletion caused by CRISPR-Cas9 decreases the proliferation of HepG2 cells, induces apoptosis, and initiates autophagy, along with G2/M arrest and disrupts cell division processes. Additionally, it inhibits EMT, lowers AURKA and CDK1/2, and inhibits migration, indicating that BIRC5 is a promising avenue for therapy for HCC.

## Supplementary Information

Below is the link to the electronic supplementary material.

Supplementary file1 (DOCX 1853 KB)

## Data Availability

No datasets were generated or analysed during the current study.

## Declarations

### Permission to publish

Each author made a contribution to the collection and analysis of the data. The final version of the text has been reviewed and approved by all authors, who have given their agreement for publication.

## References

[CR1] Alagoz M, Kherad N (2020) Advance genome editing technologies in the treatment of human diseases: CRISPR therapy (Review). Int J Mol Med 46(2):521–534. 10.3892/IJMM.2020.4609/HTML 32467995 10.3892/ijmm.2020.4609 PMC7307811

[CR2] Albadari N, Li W (2023) Survivin small molecules inhibitors : recent advances 10.3390/molecules28031376 PMC9919791 36771042

[CR3] Altieri DC (2015) Survivin - the inconvenient IAP. Semin Cell Dev Biol 39:91–96. 10.1016/j.semcdb.2014.12.007 25591986 10.1016/j.semcdb.2014.12.007 PMC4410054

[CR4] Anwanwan D, Singh SK, Singh S, Saikam V, Singh R (2020) Challenges in liver cancer and possible treatment approaches. Biochim Biophys Acta Rev Cancer 1873(1):188314. 10.1016/j.bbcan.2019.188314 31682895 10.1016/j.bbcan.2019.188314 PMC6981221

[CR5] Bray F et al (2024) Global cancer statistics 2022: GLOBOCAN estimates of incidence and mortality worldwide for 36 cancers in 185 countries. CA Cancer J Clin 74(3):229–263. 10.3322/caac.21834 38572751 10.3322/caac.21834

[CR6] Cabral LKD, Tiribelli C, Sukowati CHC (2020) Sorafenib resistance in hepatocellular carcinoma: the relevance of genetic heterogeneity. Cancers (Basel) 12(6):1–19. 10.3390/cancers12061576 10.3390/cancers12061576 PMC7352671 32549224

[CR7] Cai H, Xie X, Ji L, Ruan X, Zheng Z (2017) Sphingosine kinase 1: A novel independent prognosis biomarker in hepatocellular carcinoma. Oncol Lett 13(4):2316–2322. 10.3892/OL.2017.5732 28454397 10.3892/ol.2017.5732 PMC5403457

[CR8] Chandele A, Prasad V, Jagtap JC, Shukla R, Shastry PR (2004) Upregulation of survivin in G2/M cells and inhibition of caspase 9 activity enhances resistance in staurosporine-induced apoptosis. Neoplasia 6(1):29–40. 10.1016/s1476-5586(04)80051-4 15068669 10.1016/s1476-5586(04)80051-4 PMC1679816

[CR9] Chen Y, Jiang Q, Xue Y, Chen W, Hua M (2024) CRISPR-Cas9-mediated deletion enhancer of MECOM play a tumor suppressor role in ovarian cancer. Funct Integr Genomics 24(4):1–9. 10.1007/S10142-024-01399-8/METRICS 10.1007/s10142-024-01399-8 38995475

[CR10] Cheung CHA, Chang YC, Lin TY, Cheng SM, Leung E (2020) Anti-apoptotic proteins in the autophagic world: An update on functions of XIAP, Survivin, and BRUCE. J Biomed Sci 27(1):1–10. 10.1186/s12929-020-0627-5 32019552 10.1186/s12929-020-0627-5 PMC7001279

[CR11] Chomczynski P (1993) A reagent for the single-step simultaneous isolation of RNA, DNA and proteins from cell and tissue samples. Biotechniques 15(3):532–4 7692896

[CR12] Engeland K (2022) Cell cycle regulation : p53-p21-RB signaling. (March). 10.1038/s41418-022-00988-z 10.1038/s41418-022-00988-z PMC9090780 35361964

[CR13] Feng W, Yoshida A, Ueda T (2013) YM155 induces caspase-8 dependent apoptosis through downregulation of survivin and Mcl-1 in human leukemia cells. Biochem Biophys Res Commun 435(1):52–57. 10.1016/j.bbrc.2013.04.036 23618862 10.1016/j.bbrc.2013.04.036

[CR14] Forner A, Reig M, Bruix J (2018) Hepatocellular carcinoma. The Lancet 391(10127):1301–1314. 10.1016/S0140-6736(18)30010-2 10.1016/S0140-6736(18)30010-2 29307467

[CR15] Franken NAP, Rodermond HM, Stap J, Haveman J, van Bree C (2006) Clonogenic assay of cells in vitro. Nat Protoc 1(5):2315–2319. 10.1038/nprot.2006.339 17406473 10.1038/nprot.2006.339

[CR16] Frazzi R (2021) BIRC3 and BIRC5: multi-faceted inhibitors in cancer. Cell Biosci 11(1):1–14. 10.1186/s13578-020-00521-0 33413657 10.1186/s13578-020-00521-0 PMC7792207

[CR17] Fu J et al (2021) Identification of the hub gene BUB1B in hepatocellular carcinoma via bioinformatic analysis and in vitro experiments. PeerJ 9:1–22. 10.7717/peerj.10943 10.7717/peerj.10943 PMC7908873 33665036

[CR18] Gaj T, Gersbach CA, Barbas CF (2013) ZFN, TALEN, and CRISPR/Cas-based methods for genome engineering. Trends Biotechnol 31(7):397–405. 10.1016/J.TIBTECH.2013.04.004 23664777 10.1016/j.tibtech.2013.04.004 PMC3694601

[CR19] Haglid KG, Hamberger A, Hansson HA, HydéN H, Persson L, Rönnbäck L (1975) Control of apoptosis and mitotic spindle checkpoint by survivin. Nature 258(5537):748–749. 10.1038/258748a0 1107854 10.1038/258748a0

[CR20] Han S et al (2015) Knock out CD44 in reprogrammed liver cancer cell C3A increases CSCs stemness and promotes differentiation. Oncotarget 6(42):44452–44465. 10.18632/ONCOTARGET.6090 26540347 10.18632/oncotarget.6090 PMC4792568

[CR21] He J, Zhang, Li A, Chen F, R L 3 (2018) Knockout of NCOA5 impairs proliferation and migration of hepatocellular carcinoma cells by suppressing epithelial-to-mesenchymal transition. Biochem Biophys Res Communicationsrch 500(2):177–183 10.1016/j.bbrc.2018.04.017 29626478

[CR22] Huang X, Wu Z, Mei Y, Wu M (2013) XIAP inhibits autophagy via XIAP-Mdm2-p53 signalling. 32(16):2204–2216. 10.1038/emboj.2013.133 10.1038/emboj.2013.133 PMC3746193 23749209

[CR23] Ito T et al (2000) Survivin promotes cell proliferation in human hepatocellular carcinoma. Hepatology 31(5):1080–1085. 10.1053/he.2000.6496 10796883 10.1053/he.2000.6496

[CR24] Iwagami Y et al (2016) Aspartate β-hydroxylase modulates cellular senescence through glycogen synthase kinase 3β in hepatocellular carcinoma. Hepatology 63(4):1213–1226. 10.1002/HEP.28411/SUPPINFO 26683595 10.1002/hep.28411 PMC4805474

[CR25] Jiang C, Meng L, Yang B, Luo X (2020) Application of CRISPR/Cas9 gene editing technique in the study of cancer treatment. Clin Genet 97(1):73–88. 10.1111/cge.13589 31231788 10.1111/cge.13589

[CR26] Kamran M et al (2017) Aurora kinase A regulates Survivin stability through targeting FBXL7 in gastric cancer drug resistance and prognosis. Oncogenesis 6(2). 10.1038/oncsis.2016.80 10.1038/oncsis.2016.80 PMC5337621 28218735

[CR27] Khan Z, Arif A, Prasad GBKS, Khan N, Pramod R, Singh P (2016) Growth inhibition and chemo-radiosensitization of head and neck squamous cell carcinoma ( HNSCC ) by survivin-siRNA lentivirus. Radiother Oncol 118(2):359–368. 10.1016/j.radonc.2015.12.007 26747757 10.1016/j.radonc.2015.12.007

[CR28] Kondapuram SK, Ramachandran HK, Arya H, Coumar MS (2023) Targeting survivin for cancer therapy: strategies, small molecule inhibitors and vaccine based therapeutics in development. Life Sci 335(October):122260. 10.1016/j.lfs.2023.122260 37963509 10.1016/j.lfs.2023.122260

[CR29] Kurien BT, Scofield RH (2003) Protein blotting: a review. J Immunol Methods 274(1–2):1–15. 10.1016/S0022-1759(02)00523-9 12609528 10.1016/s0022-1759(02)00523-9

[CR30] Li Y, Lu W, Yang J, Edwards M, Jiang S (2021) Survivin as a biological biomarker for diagnosis and therapy. Expert Opin Biol Ther 21(11):1429–1441. 10.1080/14712598.2021.1918672 33877952 10.1080/14712598.2021.1918672

[CR31] Liang CC, Park AY, Guan JL (2007) In vitro scratch assay: a convenient and inexpensive method for analysis of cell migration in vitro. Nat Protoc 2(2):329–333. 10.1038/nprot.2007.30 17406593 10.1038/nprot.2007.30

[CR32] Lin T et al (2020) BIRC5 / Survivin is a novel ATG12 – ATG5 conjugate interactor and an autophagy-induced DNA damage suppressor in human cancer and mouse embryonic fibroblast cells. Autophagy 16(7):1296–1313. 10.1080/15548627.2019.1671643 31612776 10.1080/15548627.2019.1671643 PMC7469615

[CR33] Ling X, Cao S, Cheng Q, Keefe JT, Rustum YM, Li F (2012) A novel small molecule FL118 that selectively inhibits survivin, Mcl-1, XIAP and cIAP2 in a p53-independent manner, shows superior antitumor activity. PLoS One 7(9). 10.1371/journal.pone.0045571 10.1371/journal.pone.0045571 PMC3446924 23029106

[CR34] Liu W, Zhu F, Jiang Y, Sun D, Yang B, Yan H (2013) siRNA targeting survivin inhibits the growth and enhances the chemosensitivity of hepatocellular carcinoma cells. Oncol Rep 29(3):1183–1188. 10.3892/or.2012.2196 23254641 10.3892/or.2012.2196

[CR35] Liu LP, Chen JK, Liu YM, Zhang DH, Zhang J, Yang XL (2017) Depletion of GP73 inhibits invasion and metastasis of hepatocellular carcinoma cells. Zhonghua Zhong Liu Za Zhi 39(7):497–501. 10.3760/CMA.J.ISSN.0253-3766.2017.07.004 28728294 10.3760/cma.j.issn.0253-3766.2017.07.004

[CR36] Liu L, Liao J, He X, Li P (2017) The role of autophagy in hepatocellular carcinoma : Friend or foe 10.18632/oncotarget.17202 PMC5593678 28915706

[CR37] Livak KJ, Schmittgen TD (2001) Analysis of relative gene expression data using real-time quantitative PCR and the 2(-delta delta C(T)) method. Methods 25(4):402–408. 10.1006/METH.2001.1262 11846609 10.1006/meth.2001.1262

[CR38] Lone BA, Karna SKL, Ahmad F, Shahi N, Pokharel YR (2018) CRISPR/Cas9 System: A bacterial tailor for genomic engineering. Genet Res Int 2018. 10.1155/2018/3797214 10.1155/2018/3797214 PMC6167567 30319822

[CR39] Martínez-García D, Manero-Rupérez N, Quesada R, Korrodi-Gregório L, Soto-Cerrato V (2019) Therapeutic strategies involving survivin inhibition in cancer. Med Res Rev 39(3):887–909. 10.1002/med.21547 30421440 10.1002/med.21547

[CR40] Mi N et al (2020) Identification of hub genes involved in the occurrence and development of hepatocellular carcinoma via bioinformatics analysis. Oncol Lett 20(2):1695–1708. 10.3892/ol.2020.11752 32724412 10.3892/ol.2020.11752 PMC7377146

[CR41] Mohamed NM, Mohamed RH, Kennedy JF, Elhefnawi MM, Hamdy NM (2025) A comprehensive review and in silico analysis of the role of survivin (BIRC5) in hepatocellular carcinoma hallmarks: A step toward precision, Int J Biol Macromolecules, Volume 311. Part 1. 10.1016/j.ijbiomac.2025.143616 10.1016/j.ijbiomac.2025.143616 40306500

[CR42] Narimani M et al (2019) BIRC5 gene disruption via CRISPR/cas9n platform suppress acute myelocytic leukemia progression. Iran Biomed J 23(6):369–378. 10.29252/ibj.23.6.369 31104397 10.29252/ibj.23.6.369 PMC6800533

[CR43] Niu J, Zhang B, Chen H (2014) Applications of TALENs and CRISPR/Cas9 in Human Cells and Their Potentials for Gene Therapy. Mol Biotechnol 56(8):681–688. 10.1007/s12033-014-9771-z 24870618 10.1007/s12033-014-9771-z

[CR44] Peery RC, Liu JY, Zhang JT (2017) Targeting survivin for therapeutic discovery: past, present, and future promises. Drug Discov Today 22(10):1466–1477. 10.1016/j.drudis.2017.05.009 28577912 10.1016/j.drudis.2017.05.009

[CR45] Peng Y, Wang Y, Zhou C, Mei W, Zeng C (2022) PI3K/Akt/mTOR pathway and its role in cancer therapeutics: are we making headway? Front Oncol 12(March):1–17. 10.3389/fonc.2022.819128 10.3389/fonc.2022.819128 PMC8987494 35402264

[CR46] Pott LL et al (2017) Eukaryotic elongation factor 2 is a prognostic marker and its kinase a potential therapeutic target in HCC. Oncotarget 8(7):11950–11962. 10.18632/ONCOTARGET.14447 28060762 10.18632/oncotarget.14447 PMC5355317

[CR47] Rabaan AA et al (2023) Application of CRISPR/Cas9 technology in cancer treatment: a future direction. Curr Oncol 30(2):1954–1976. 10.3390/curroncol30020152 36826113 10.3390/curroncol30020152 PMC9955208

[CR48] Rajanathadurai J, Perumal E, Sindya J (2024) Advances in targeting cancer epigenetics using CRISPR-dCas9 technology: a comprehensive review and future prospects. Funct Integr Genomics 24(5):1–18. 10.1007/S10142-024-01455-3/METRICS 10.1007/s10142-024-01455-3 39292321

[CR49] Rao DVS, Muraleedharan K, Humphreys CJ (2010) TEM specimen preparation techniques. (320):1232–1244

[CR50] Rashed WM, Kandeil MAM, Mahmoud MO, Ezzat S (2020) Hepatocellular Carcinoma (HCC) in Egypt: a comprehensive overview. J Egypt Natl Canc Inst 32(1). 10.1186/s43046-020-0016-x 10.1186/s43046-020-0016-x PMC13325438 32372179

[CR51] Rawal P (n.d.) Targeted HBx gene editing by CRISPR / Cas9 system effectively reduces epithelial to mesenchymal transition and HBV replication in hepatoma cells, pp 1–16 10.1111/liv.15805 38105495

[CR52] Song J et al (2018) CRISPR/Cas9-mediated knockout of HBsAg inhibits proliferation and tumorigenicity of HBV-positive hepatocellular carcinoma cells. J Cell Biochem 119(10):8419–8431. 10.1002/jcb.27050 29904948 10.1002/jcb.27050 PMC6221038

[CR53] Su C (2016) Survivin in survival of hepatocellular carcinoma. Cancer Lett 379(2):184–190. 10.1016/j.canlet.2015.06.016 26118774 10.1016/j.canlet.2015.06.016

[CR54] Suzuki A et al (2000) Survivin initiates cell cycle entry by the competitive interaction with Cdk4/p16(INK4a) and Cdk2/Cyclin E complex activation. Oncogene 19(29):3225–3234. 10.1038/sj.onc.1203665 10918579 10.1038/sj.onc.1203665

[CR55] Tai C, Chin-sheng H, Kuo L, Wei P (2012) Survivin-mediated cancer cell migration through GRP78 and epithelial-mesenchymal transition ( EMT ) marker expression in mahlavu cells 336–343. 10.1245/s10434-011-1692-5 10.1245/s10434-011-1692-5 21516372

[CR56] Vogel A, Saborowski A (2020) Current strategies for the treatment of intermediate and advanced hepatocellular carcinoma. Cancer Treat Rev 82:101946. 10.1016/j.ctrv.2019.101946 31830641 10.1016/j.ctrv.2019.101946

[CR57] Wang Q, Chen Z, Diao X, Huang S (2011) Induction of autophagy-dependent apoptosis by the survivin suppressant YM155 in prostate cancer cells. Cancer Lett 302(1):29–36. 10.1016/j.canlet.2010.12.007 21220185 10.1016/j.canlet.2010.12.007

[CR58] Wang X, Zhang W, Ding Y, Guo X, Yuan Y, Li D (2017) CRISPR/Cas9-mediated genome engineering of CXCR4 decreases the malignancy of hepatocellular carcinoma cells in vitro and in vivo. Oncol Rep 37(6):3565–3571. 10.3892/or.2017.5601 28498420 10.3892/or.2017.5601

[CR59] Wang Z, Fukuda S, Pelus LM (2004) Survivin regulates the p53 tumor suppressor gene family. (September):8146–8153. 10.1038/sj.onc.1207992 10.1038/sj.onc.1207992 15361831

[CR60] Wang D, Liu J, Liu S, Li W (2020) Identification of crucial genes associated with immune cell infiltration in hepatocellular carcinoma by weighted gene co-expression network analysis. 11(April):1–17. 10.3389/fgene.2020.00342 10.3389/fgene.2020.00342 PMC7193721 32391055

[CR61] Wang H, Guo M, Wei H, Chen Y (2023) Targeting p53 pathways : mechanisms , structures , and advances in therapy. (February):1–35. 10.1038/s41392-023-01347-1 10.1038/s41392-023-01347-1 PMC9977964 36859359

[CR62] Wei L et al (2017) Histone methyltransferase G9a promotes liver cancer development by epigenetic silencing of tumor suppressor gene RARRES3. J Hepatol 67(4):758–769. 10.1016/J.JHEP.2017.05.015 28532996 10.1016/j.jhep.2017.05.015

[CR63] Wheatley SP, Altieri DC (2019) Survivin at a glance. J Cell Sci 132(7). 10.1242/jcs.223826 10.1242/jcs.223826 PMC6467487 30948431

[CR64] Xie F, Wang J, Zhang B (2023) RefFinder: a web-based tool for comprehensively analyzing and identifying reference genes. Funct Integr Genomics 23(2):1–5. 10.1007/S10142-023-01055-7/METRICS 10.1007/s10142-023-01055-7 37060478

[CR65] Yang X, Zhang B (2023) A review on CRISPR/Cas: a versatile tool for cancer screening, diagnosis, and clinic treatment. Funct Integr Genomics 23(2):1–26. 10.1007/S10142-023-01117-W/METRICS 10.1007/s10142-023-01117-w 37231285

[CR66] Yang P, Wang G, Huo H, Li Q, Zhao Y, Liu Y (2015) SDF-1/CXCR4 signaling up-regulates survivin to regulate human sacral chondrosarcoma cell cycle and epithelial–mesenchymal transition via ERK and PI3K/AKT pathway. Med Oncol 32(1):1–7. 10.1007/s12032-014-0377-x 10.1007/s12032-014-0377-x 25428386

[CR67] Yu J, Wang Z, Zhang H, Wang Y, Li DQ (2021) Survivin-positive circulating tumor cells as a marker for metastasis of hepatocellular carcinoma. World J Gastroenterol 27(43):7546–7562. 10.3748/wjg.v27.i43.7546 34887648 10.3748/wjg.v27.i43.7546 PMC8613743

[CR68] Zhang Z et al (2017) Small interfering RNA targeting of the survivin gene inhibits human tumor cell growth in vitro. Exp Ther Med 14(1):35–42. 10.3892/etm.2017.4501 28672890 10.3892/etm.2017.4501 PMC5488478

[CR69] Zhang L et al (2019) The clinical significance of endothelin receptor type B in hepatocellular carcinoma and its potential molecular mechanism. Exp Mol Pathol 107(July 2018):141–157. 10.1016/j.yexmp.2019.02.002 30768923 10.1016/j.yexmp.2019.02.002

[CR70] Zhang S, Zhang F, Chen Q, Wan C, Xiong J, Xu J (2019) CRISPR/Cas9-mediated knockout of NSD1 suppresses the hepatocellular carcinoma development via the NSD1/H3/Wnt10b signaling pathway. J Exp Clin Cancer Res 38(1):1–14. 10.1186/s13046-019-1462-y 31727171 10.1186/s13046-019-1462-y PMC6854717

[CR71] Zhao G et al (2017) Lentiviral CRISPR/Cas9 nickase vector mediated BIRC5 editing inhibits epithelial to mesenchymal transition in ovarian cancer cells. Oncotarget 8(55):94666–94680. 10.18632/oncotarget.21863 29212257 10.18632/oncotarget.21863 PMC5706903

[CR72] Zhou SJ, Deng YL, Liang HF, Jaoude JC, Liu FY (2017) Hepatitis B virus X protein promotes CREB-mediated activation of miR-3188 and Notch signaling in hepatocellular carcinoma. Cell Death Differ 24(9):1577. 10.1038/CDD.2017.87 28574502 10.1038/cdd.2017.87 PMC5563993

