# Supplementary material for: BIRC5 as a master regulator in HCC: unraveling its role in tumor survival and therapeutic potential
Source: Funct Integr Genomics. 2025 Jun 5;25(1):120. doi: 10.1007/s10142-025-01615-z (PMC12141170; doi:10.1007/s10142-025-01615-z)
Supplement: Supplementary file 1 — Supplementary file1 (DOCX 1853 KB) [file 10142_2025_1615_MOESM1_ESM.docx]

# Supplementary information

**
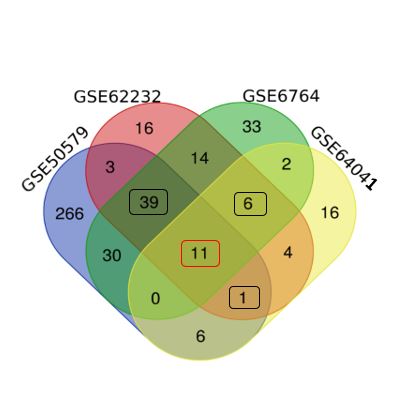
Fig. S1**: Venn diagram showing the significantly upregulated common genes identified from GSE50579, GSE62232, GSE6764 and GSE64041 HCC microarray datasets.

| **a** 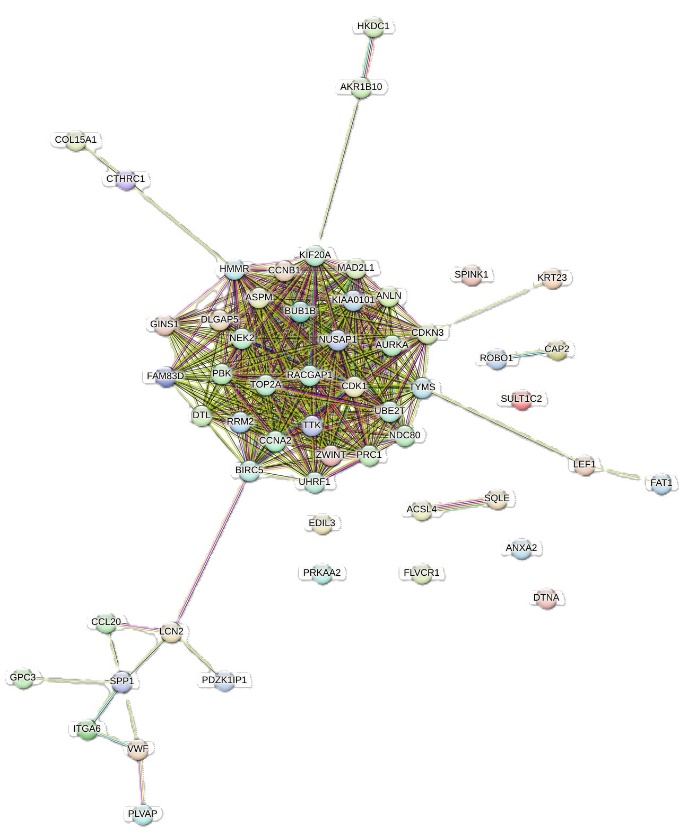 | 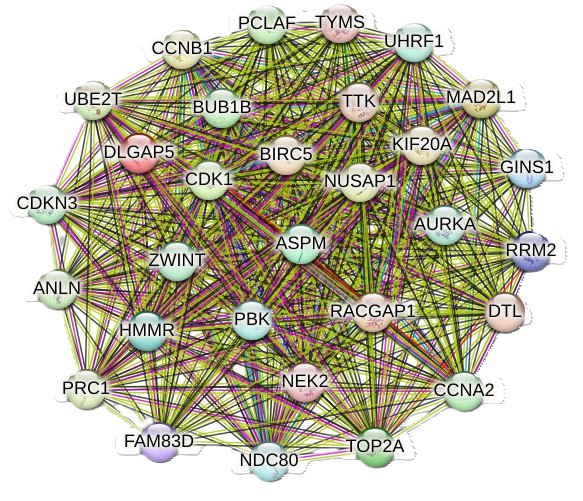**b** |
| --- | --- |

**Fig. S2: PPI network construction and module analysis**. a) PPI network was constructed based on the 57 genes using the STRING database. Module analysis of the PPI network was performed by the MCODE plugin of Cytoscape. b) PPI network construction of Thirty central nodes. Colored nodes represent the production of common upregulated genes. Edges represent the protein-protein associations. PPI= Protein-protein interaction.

| 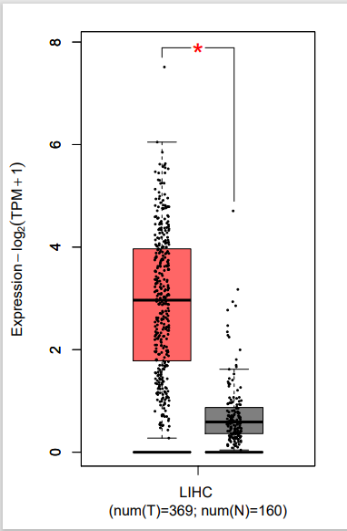**a** BIRC5 | 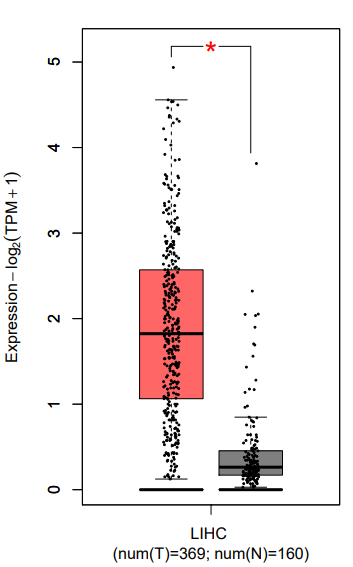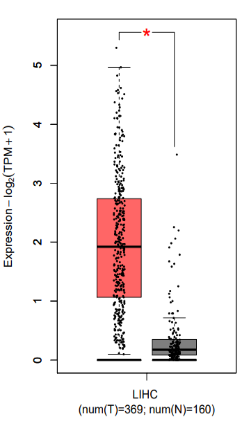 HMMR KIF20A | |
| --- | --- | --- |
| **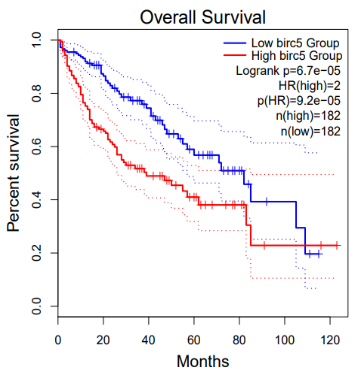b** BIRC5 | 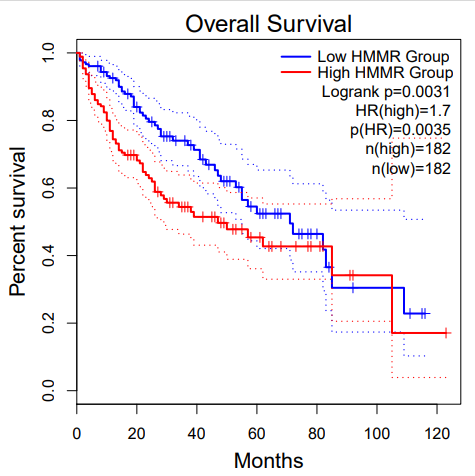 HMMR | 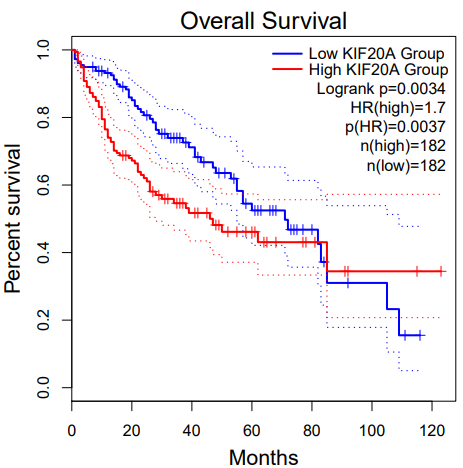 KIF20A |

**Fig. S3. GEPIA analysis of the three top hub genes with degree 60. a**) Box plots of BIRC5, HMMR and KIF20A mRNA expression levels. The expression level is described by log2(TPM + 1). These box plots compared 369 HCC samples (red) with 160 normal liver samples (gray). *P < 0.05 was considered statistically significant. **b)** Overall survival of BIRC5, HMMR and KIF20A in patients with HCC. The data are presented as the hazard ratios (HR). Log–rank P < 0.01 was regarded as statistically significant. TPM, transcript count per million; HCC, hepatocellular carcinoma; LIHC, liver hepatocellular carcinoma.

Table (1): KEGG Pathway and Gene Ontology analysis of the upregulated genes in HCC microarray datasets.

| Category | Term | Count | P-Value | Benjamini |
| --- | --- | --- | --- | --- |
| KEGG_PATHWAY | Cell cycle | 7 | 1.40E-05 | 1.60E-03 |
| KEGG_PATHWAY | Progesterone-mediated oocyte maturation | 5 | 4.10E-04 | 2.30E-02 |
| KEGG_PATHWAY | ECM-receptor interaction | 4 | 3.60E-03 | 1.30E-01 |
| KEGG_PATHWAY | Oocyte meiosis | 4 | 1.00E-02 | 2.90E-01 |
| KEGG_PATHWAY | p53 signaling pathway | 3 | 2.70E-02 | 6.20E-01 |

| GOTERM_BP_DIRECT | cell division | 13 | 4.40E-10 | 2.60E-07 |
| --- | --- | --- | --- | --- |
| GOTERM_BP_DIRECT | mitotic spindle assembly checkpoint | 6 | 2.40E-08 | 7.00E-06 |
| GOTERM_BP_DIRECT | mitotic spindle organization | 6 | 5.10E-07 | 1.00E-04 |
| GOTERM_BP_DIRECT | mitotic sister chromatid segregation | 5 | 3.40E-06 | 5.10E-04 |
| GOTERM_BP_DIRECT | chromosome segregation | 6 | 6.30E-06 | 7.50E-04 |
| GOTERM_BP_DIRECT | G2/M transition of mitotic cell cycle | 5 | 1.90E-05 | 1.90E-03 |
| GOTERM_CC_DIRECT | Spindle | 11 | 5.10E-12 | 8.50E-10 |
| GOTERM_CC_DIRECT | Midbody | 10 | 1.40E-09 | 1.10E-07 |
| GOTERM_CC_DIRECT | Kinetochore | 8 | 2.10E-07 | 1.20E-05 |
| GOTERM_CC_DIRECT | Mitotic spindle | 7 | 2.10E-06 | 8.70E-05 |
| GOTERM_CC_DIRECT | Cytoplasm | 31 | 2.00E-05 | 6.50E-04 |
| GOTERM_MF_DIRECT | microtubule binding | 7 | 1.20E-04 | 2.10E-02 |
| GOTERM_MF_DIRECT | protein serine/threonine kinase activity | 7 | 9.70E-04 | 8.30E-02 |
| GOTERM_MF_DIRECT | protein binding | 47 | 1.90E-03 | 1.10E-01 |
| GOTERM_MF_DIRECT | ATP binding | 12 | 3.60E-03 | 1.20E-01 |

BP: biological processes, CC: cell components, (MF) molecular function

Table (2): List of the hub genes identified in HCC microarray datasets

| **Name** | **Degree** | **ClosenessCentrality** | **BetweennessCentrality** | **NeighborhoodConnectivity** |
| --- | --- | --- | --- | --- |
| BIRC5 | 60 | 0.666666667 | 0.305016337 | 27.7 |
| HMMR | 60 | 0.611111111 | 0.089371516 | 27.63333333 |
| KIF20A | 60 | 0.611111111 | 0.089371516 | 27.63333333 |
| ANLN | 58 | 0.594594595 | 5.77E-04 | 28.55172414 |
| ASPM | 58 | 0.594594595 | 5.77E-04 | 28.55172414 |
| AURKA | 58 | 0.594594595 | 5.77E-04 | 28.55172414 |
| BUB1B | 58 | 0.594594595 | 5.77E-04 | 28.55172414 |
| CCNA2 | 58 | 0.594594595 | 5.77E-04 | 28.55172414 |
| CCNB1 | 58 | 0.594594595 | 5.77E-04 | 28.55172414 |
| CDK1 | 58 | 0.594594595 | 5.77E-04 | 28.55172414 |
| DLGAP5 | 58 | 0.594594595 | 5.77E-04 | 28.55172414 |
| MAD2L1 | 58 | 0.594594595 | 5.77E-04 | 28.55172414 |
| NEK2 | 58 | 0.594594595 | 5.77E-04 | 28.55172414 |
| NUSAP1 | 58 | 0.594594595 | 5.77E-04 | 28.55172414 |
| PBK | 58 | 0.594594595 | 5.77E-04 | 28.55172414 |
| PCLAF | 58 | 0.594594595 | 5.77E-04 | 28.55172414 |
| RACGAP1 | 58 | 0.594594595 | 5.77E-04 | 28.55172414 |
| RRM2 | 58 | 0.594594595 | 5.77E-04 | 28.55172414 |
| TOP2A | 58 | 0.594594595 | 5.77E-04 | 28.55172414 |
| TTK | 58 | 0.594594595 | 5.77E-04 | 28.55172414 |
| TYMS | 58 | 0.602739726 | 0.089015152 | 27.79310345 |
| UHRF1 | 58 | 0.594594595 | 5.77E-04 | 28.55172414 |
| ZWINT | 58 | 0.594594595 | 5.77E-04 | 28.55172414 |

BIRC5 (baculoviral IAP repeat containing 5), HMMR (hyaluronan mediated motility receptor), KIF20A (kinesin family member 20A), ANLN (anillin, actin binding protein), ASPM (assembly factor for spindle microtubules), AURKA (aurora kinase A), BUB1B (mitotic checkpoint serine/threonine kinase B), CCNA2 (cyclin A2), CCNB1 (cyclin B1), CDK1 (cyclin dependent kinase 1), DLGAP5 (DLG associated protein 5), MAD2L1 (mitotic arrest deficient 2 like 1), NEK2 (NIMA related kinase 2), NUSAP1 (nucleolar and spindle associated protein 1), PBK (PDZ binding kinase), PCLAF (PCNA clamp associated factor), RACGAP1 (Rac GTPase activating protein 1), RRM2 (ribonucleotide reductase M2), TOP2A (topoisomerase (DNA) II alpha 170kDa), TTK (TTK protein kinase), TYMS (thymidylate synthetase), UHRF1 (ubiquitin-like with PHD and ring finger domains 1), ZWINT (ZW10 interactor).

**Original blots and images.**

**Western blot images**

| Two independent experiments  First experiment | | |
| --- | --- | --- |
| BIRC5 (membrane 1)  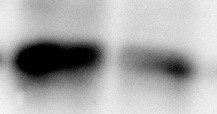 untreated eCas-BIRC5-gRNA | GAPDH (membrane 2)  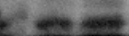 untreated eCas-BIR-gRNA | |
| Second experiment | | |
| 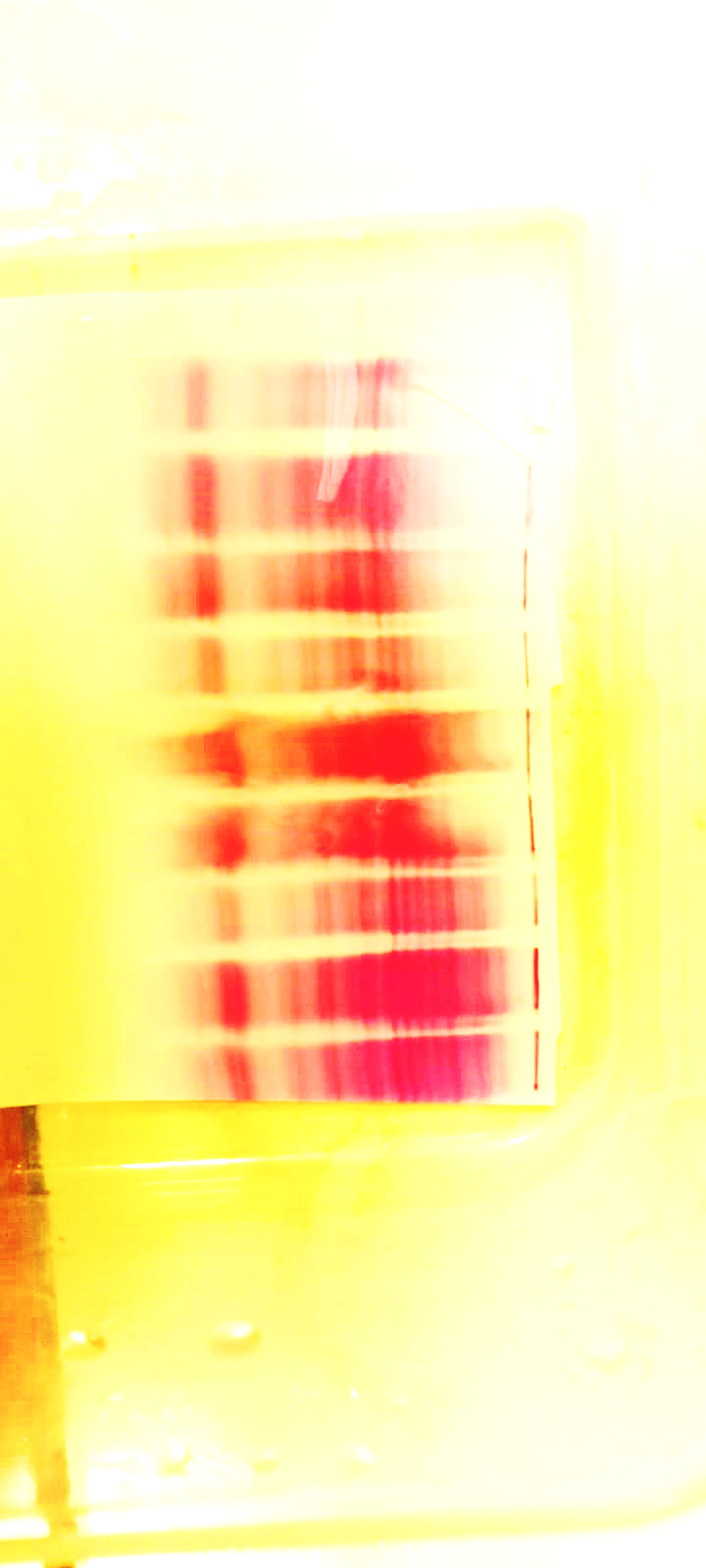BIRC5 (membrane 3)  untreated eCas-BIR-gRNA | | GAPDH (membrane 4)  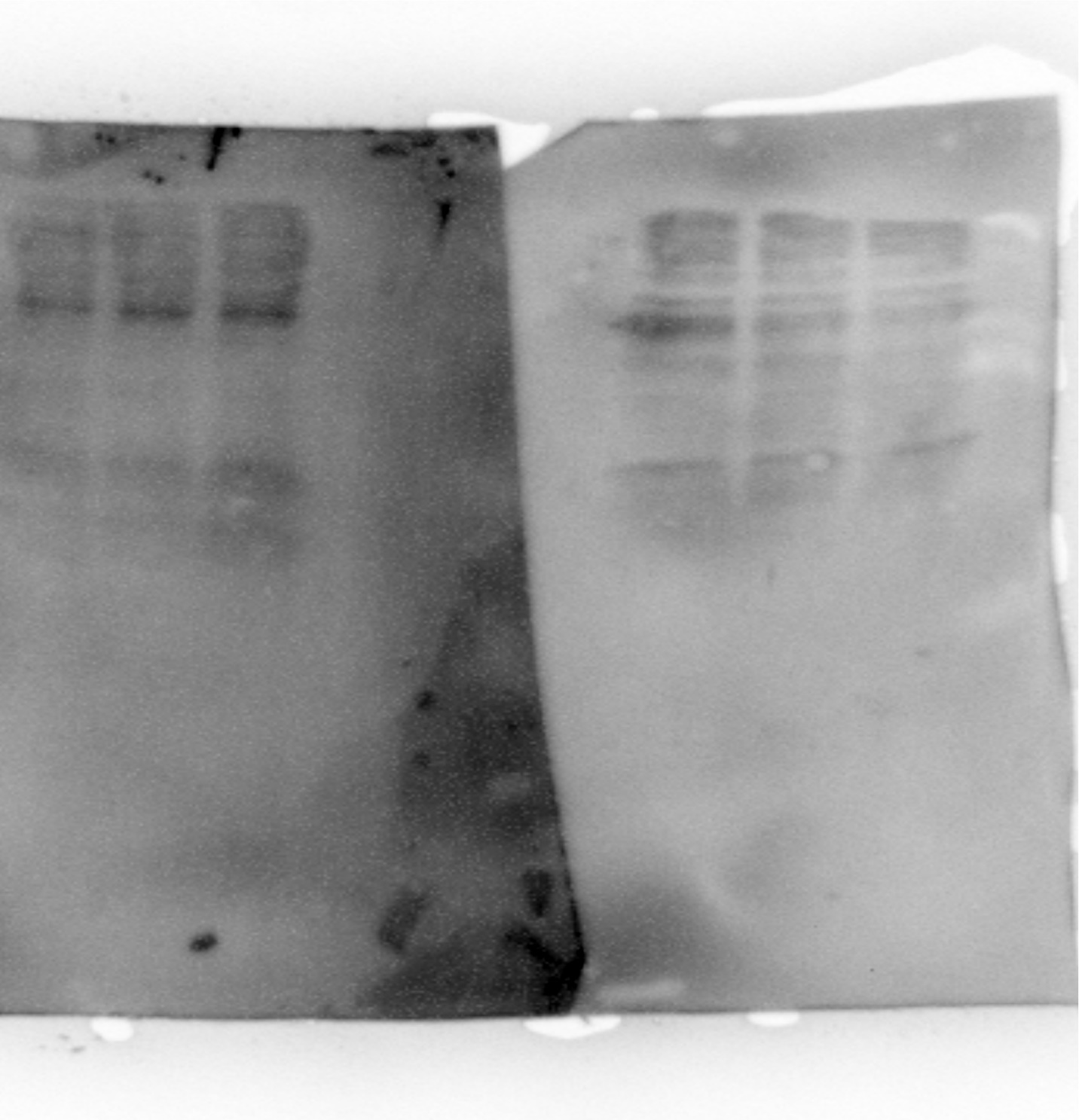 untreated eCas-BIR-gRNA |
|  | | |

| Membrane of first experiment  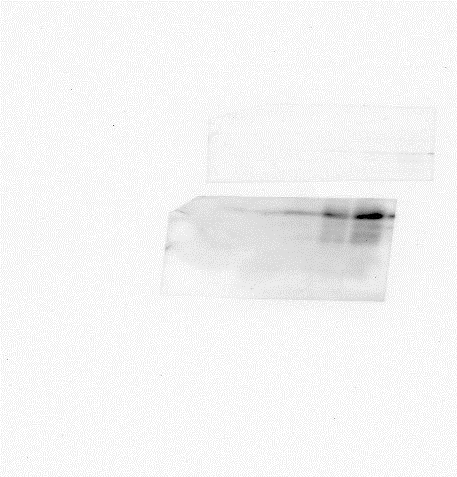**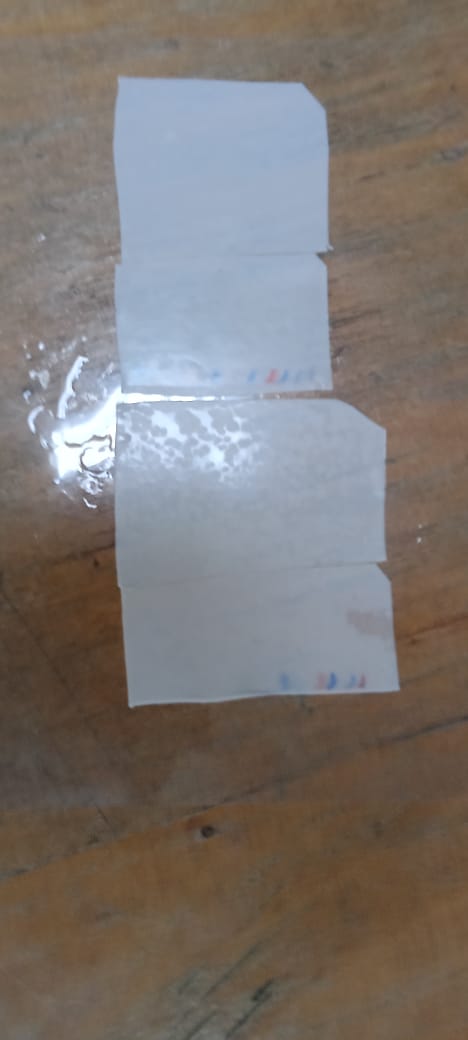**Membrane (1)  Membrane (2)  Untreated eCas-BIR-gRNA  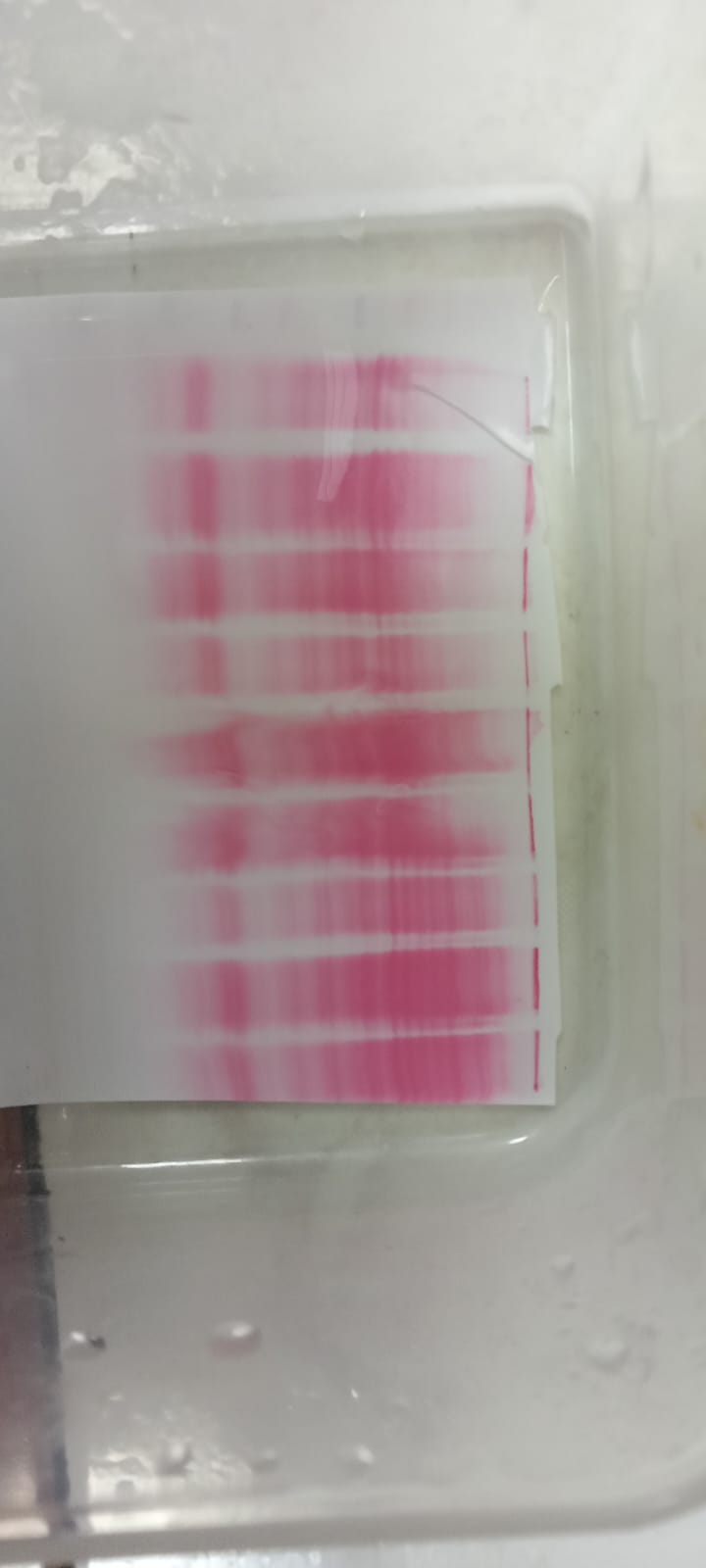  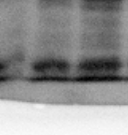 |
| --- |
|  |

| 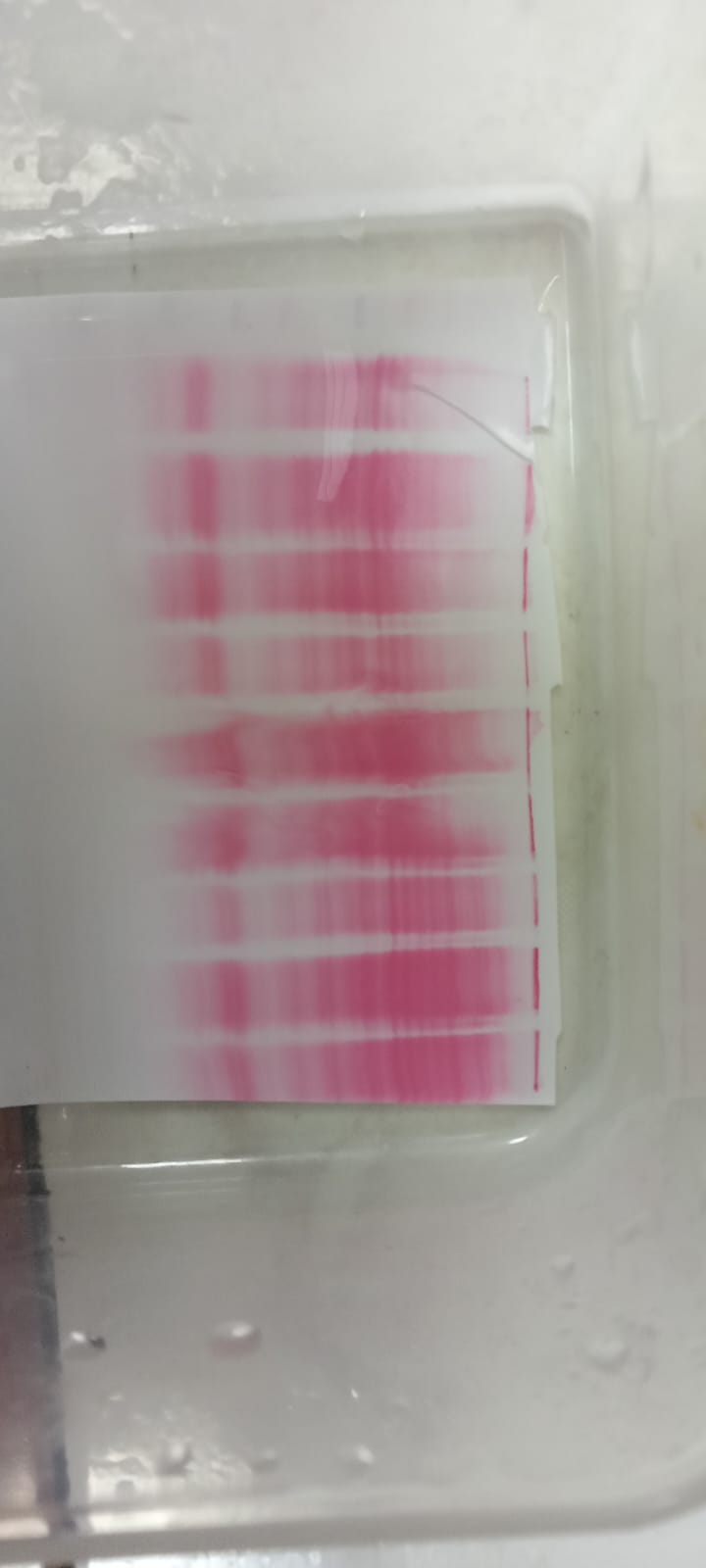Membrane 3    Marker  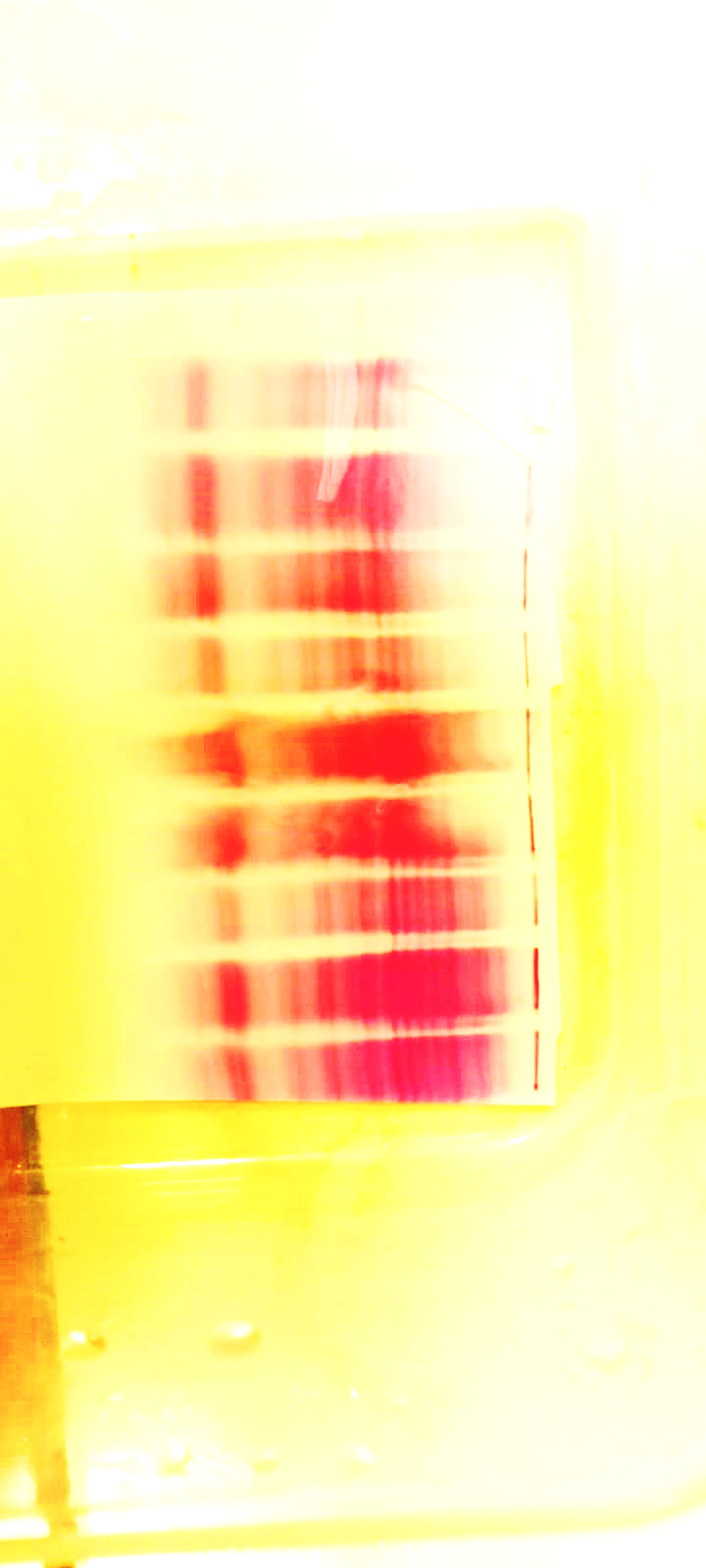  1 2 3 4 5 6 7  Lane 1 & 4 & 7: eCas-BIRC5-gRNA  Lane 2 &5: eCas  Lane 3 & 6: Unreated HepG2 |
| --- |
| 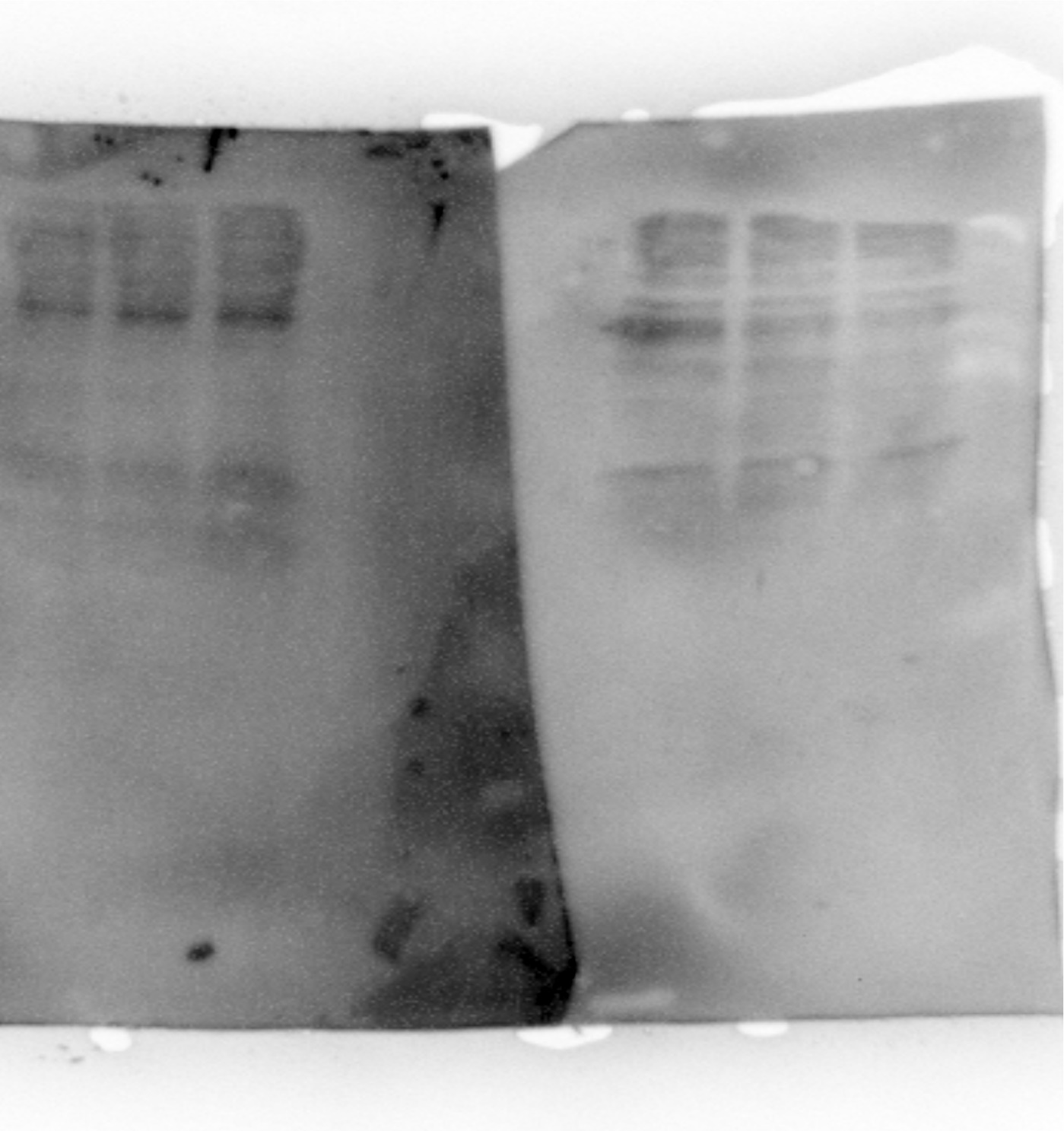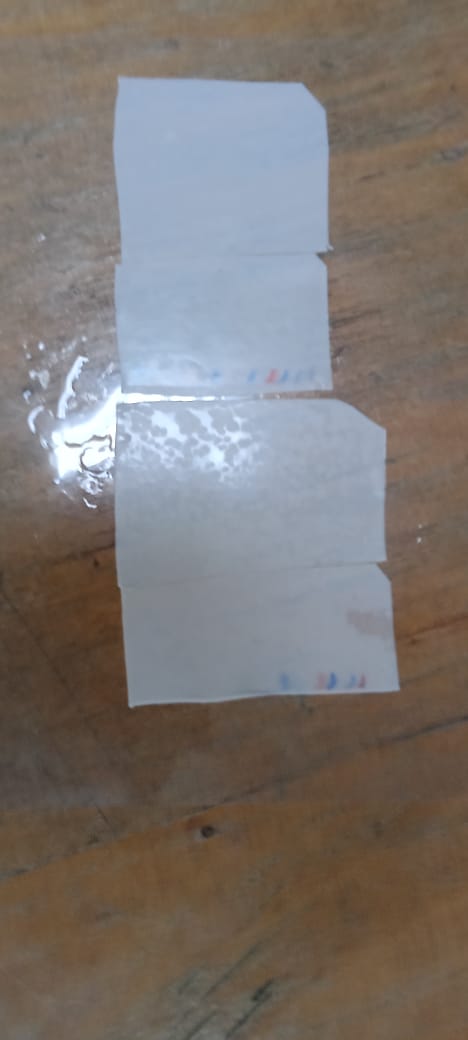Membrane 4 (GAPDH lane 1: untreated, lane 2: eCas-BIRC5-gRNA) |
| Gel (SDS gel)  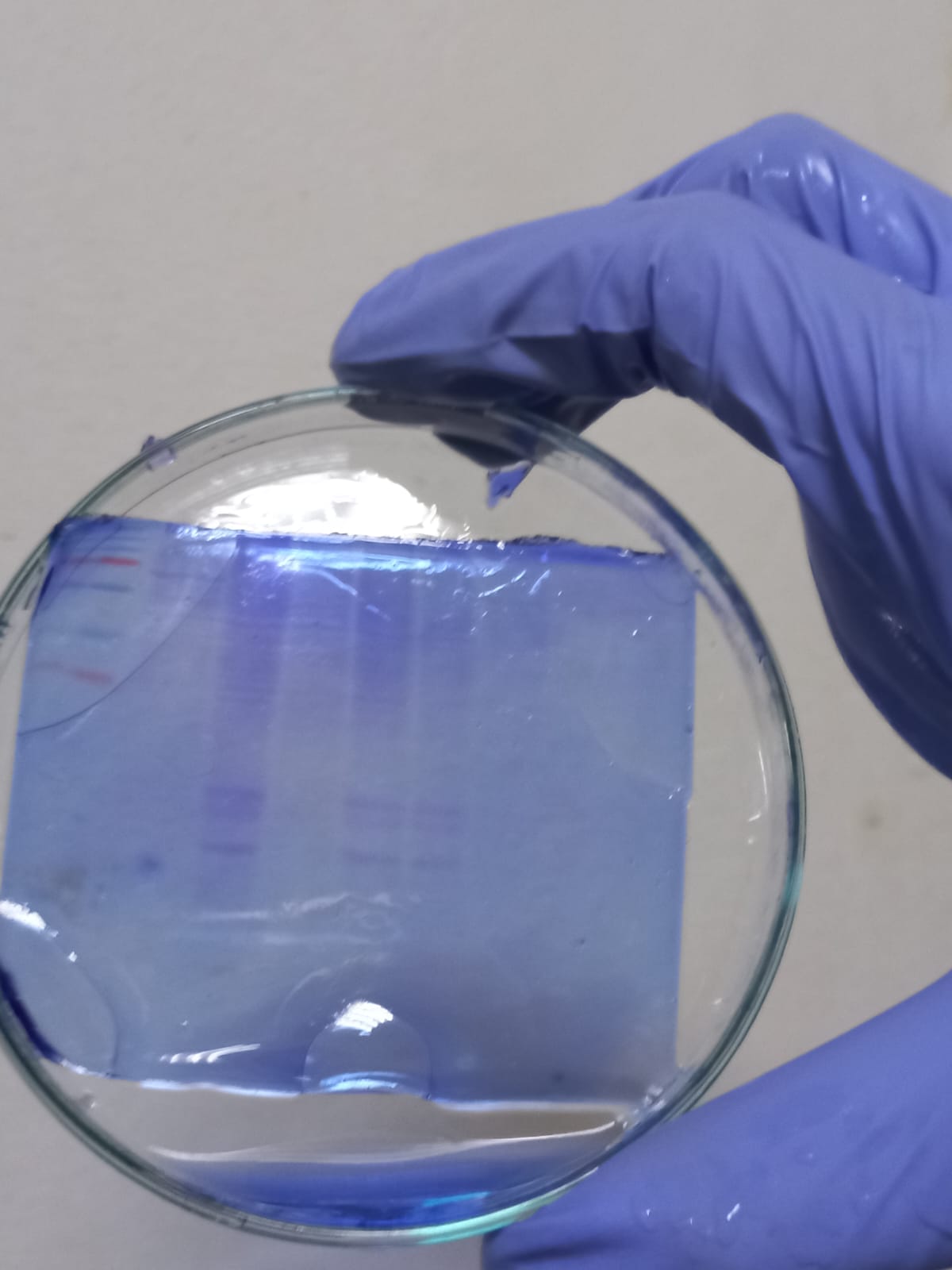 |
